# Genomic determinants of therapy response in *ETV6::RUNX1* leukemia

**DOI:** 10.1038/s41375-025-02683-7

**Published:** 2025-07-09

**Authors:** Laura Oksa, Sanni Moisio, Khurram Maqbool, Roger Kramer, Atte Nikkilä, Buddika Jayasingha, Artturi Mäkinen, Hassan Foroughi-Asl, Samuli Rounioja, Janne Suhonen, Olga Krali, Miikka Voutilainen, Mari Lahnalampi, Kaisa Vepsäläinen, Sui Huang, Jesus Duque-Afonso, Julia Hauer, Jessica Nordlund, Valtteri Wirta, Olli Lohi, Merja Heinäniemi

**Affiliations:** 1 Tampere Center for Child, Adolescent, and Maternal Health Research, Faculty of Medicine and Health Technology, Tampere University, Tampere, Finland; 2 Tampere University Hospital, Tays Cancer Centre, Tampere, Finland; 3 The Institute of Biomedicine, University of Eastern Finland, Kuopio, Finland; 4 SciLifeLab, Department of Microbiology, Tumor and Cell biology, Karolinska Institutet, Stockholm, Sweden; 5 Department of Pathology, Fimlab Laboratories, Tampere University Hospital, Tampere, Finland; 6 Genomic Medicine Center Karolinska, Karolinska University Hospital, Stockholm, Sweden; 7 Department of Hematology, Fimlab Laboratories, Tampere University Hospital, Tampere, Finland; 8 Department of Medical Sciences, Uppsala University, Uppsala, Sweden; 9 SciLifeLab, Uppsala University, Uppsala, Sweden; 10 Faculty of Biological and Environmental Sciences, University of Helsinki, Helsinki, Finland; 11 Department of Pediatrics, Kuopio University Hospital, Kuopio, Finland; 12 Institute for Systems Biology, Seattle, WA USA; 13 Department of Hematology/Oncology/Stem Cell Transplantation, Faculty of Medicine, University of Freiburg Medical Center, Freiburg, Germany; 14 Department of Pediatrics, School of Medicine, Technical University of Munich, Munich, Germany; 15 German Center for Child and Adolescent Health (DZKJ) and German Cancer Consortium (DKTK), partner site Munich, Munich, Germany

**Keywords:** Acute lymphocytic leukaemia, Cancer genetics

## Abstract

*ETV6::RUNX1* leukemia is the second most common subtype of childhood B cell acute lymphoblastic leukemia (B-ALL). Although it generally has a low relapse risk, a significant proportion of B-ALL relapses occur within this subtype due to its relatively high incidence. Measurable residual disease at the end of induction therapy is a well-established biomarker predicting treatment outcomes, while no genomic biomarkers are routinely applied in clinics. In this study, we used multiomic data from *ETV6::RUNX1* leukemias to identify genomic features predictive of therapy response at disease presentation. In the deeply characterized sub-cohort we discovered that fast-responding cases frequently exhibited the APOBEC mutational signature and the gene expression signature of high cell cycle activity. In contrast, rearrangements of *IGK* genes were more frequent in slow responders. Additionally, response-related mutations were identified in transcriptional regulators and tumor suppressor genes (*INTS1, NF1, TP53*). Copy number analysis revealed that fast responders harbored more frequent deletions of chr12 p-arm, leading to transcriptomic changes affecting genes associated with induction therapy response (*KRAS*, *FKBP4*), while a shorter gain in chr12 was more common in slow responders. The identified genetic and transcriptomic markers of treatment sensitivity pave the way for improved disease classification at presentation, potentially improving clinical outcomes.

## Introduction

*ETV6::RUNX1* is the second most frequent form of B cell acute lymphoblastic leukemia (B-ALL) in childhood. This fusion gene, arising from the chromosomal translocation t(12;21)(p13;q22), is considered the initiating event that occurs in fetal hematopoietic progenitors *in utero* [1, 2]. This subgroup generally presents with low-risk features and responds favorably to the current chemotherapy. However, approximately 5% of *ETV6::RUNX1* patients experience relapse, accounting for nearly 20% of all B-ALL relapses [3–5]. Particularly, patients with an inadequate response to induction therapy display an increased risk of relapse [6–10]. This risk can be efficiently measured using techniques that quantify measurable residual disease (MRD), which is typically assessed based on blast counts at two-time points: end of induction (EOI, around day 29) and end of consolidation (EOC, around day 79) [6, 11]. MRD is a surrogate marker that reflects underlying leukemia biology but the genetic lesions that determine therapy response remain elusive. For example, deletion of the glucocorticoid receptor *NR3C1* that confers resistance to prednisolone and dexamethasone increases the relapse risk in *ETV6::RUNX1* patients, but it only occurs in approximately half of the relapsed cases [4].

The *ETV6::RUNX1* fusion acts as a weak oncogene and is insufficient to initiate leukemia independently. Therefore, leukemia onset depends on the acquisition of additional genetic lesions that typically occur during the first years of life [1, 12, 13]. Compared to other ALL subtypes, the *ETV6::RUNX1* cells have higher recombination activating (RAG) complex activity through elevated *RAG1/2* gene expression [14, 15]. This increased activity can drive leukemogenesis by introducing structural variants (SVs) through off-target DNA breakpoints. Secondary abnormalities often include focal deletions that harbor RAG recognition motifs. In most cases, these deletions typically affect only a few genomic loci, with the most frequent target genes being *ETV6*, *CDKN2A/B*, *PAX5*, *TCF4, EBF1, CD200, TBL1XR1*, and *BTLA* [14, 16, 17]. In addition to focal deletions, gains of chromosome arms and extra chromosomes are also recurrently observed in *ETV6::RUNX1* leukemia, with an average of four copy number variations (CNVs) per case. The most common CNVs in this subtype are trisomy 21, derivative chromosome der (21)t(12;21), 12p deletions, trisomy 10, trisomy 16, and duplication of the long arm of chromosome X [14, 16–18]. Compared to other cancer types, ALL typically has a relatively low single nucleotide variant (SNV) or insertion-deletion (InDel) mutation frequency, with most mutations being passenger mutations that do not directly contribute to the selective advantage of leukemic cells [19–23]. Studies of mutational signatures in *ETV6::RUNX1*-positive ALL have demonstrated that some COSMIC-defined signatures, such as those associated with aging, APOBEC activity, or DNA repair, are enriched in ALL cells [14, 17, 24]. These mutational processes can accelerate leukemogenesis, with some signatures, such as those caused by AID/APOBEC enzymes (SBS2 and 3), and ultraviolet light (SBS7), being linked to relapse. However, the exact mechanisms and functions of these signatures in ALL remain unclear [17, 23]. The presence of recurrent secondary mutations and their unique combinations underscores the genetic diversity of this disease [16].

Herein, we studied the genomic determinants of therapy response in *ETV6::RUNX1* leukemia by integrative analysis of array-based copy number analysis (CNA), whole genome (WGS) and targeted DNA sequencing, as well as sequencing of the transcriptome, in a cohort of patients stratified based on the therapy response to contemporary induction treatment.

## Results

### Somatic mutation rate is not associated with treatment response

**Fig. 1:**
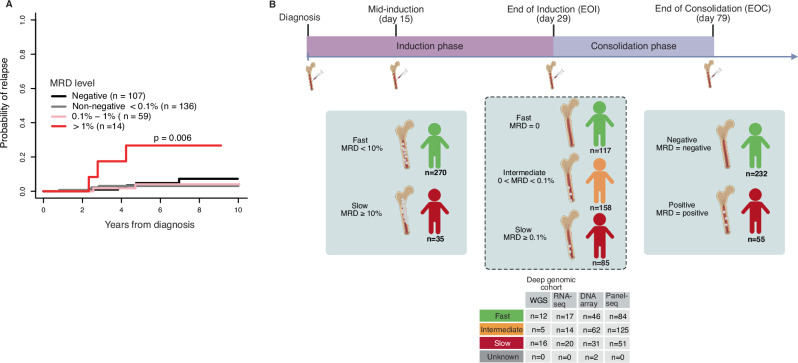
MRD-stratified analysis of the *ETV6::RUNX1* patient cohorts. **A** Cumulative incidence of relapse by MRD level at EOI in the NOPHO ALL2008 cohort (*n *= 316). **B**. Study design showing the response group classification and number of cases profiled in each genomics assay by EOI response. A subset of cases was profiled in multiple genomic analyses (refer to Fig. S1A-B).

The slow clearance of leukemic blasts has been found to be a significant predictor of relapse [9]. Accordingly, a positive correlation between high MRD at EOI and the cumulative incidence of relapse was observed in the Nordic NOPHO ALL2008 cohort of 316 *ETV6::RUNX1* cases (Fig. 1A). The increased risk of relapse was evident for patients with MRD levels above 1% after EOI, with a 26.7% (0–54%) probability of relapse five years after diagnosis. In comparison, patients with MRD levels below 1% after EOI had a significantly lower risk of relapse: 4.1% (0–9.8%) for MRD between 0.1% and 1%, 3.1% (0.1–6.0%) for MRD below 0.1%, and 4.8% (0.1–9.4%) for those with undetectable MRD. Cases with MRD levels above 0.1% at EOI received more intensified therapy (intermediate risk arm), likely masking the impact of MRD on the relapse risk. As a result, in subsequent genomic analyses, a slow EOI therapy response was defined as MRD levels greater or equal to 0.1%, intermediate response as measurable MRD at EOI (but below 0.1%), and fast response as undetectable MRD (MRD-negative) at EOI. In addition, we analyzed the response at two additional time points: mid-induction, and end of consolidation (EOC): fast responders were defined as having MRD < 10% at mid-induction, and at EOC, patients were classified as either MRD-negative (undetectable MRD) or MRD-positive (detectable MRD). The EOI response served as the primary time point for evaluating treatment outcomes (Fig. 1B, Supplementary Table 1).

To date, MRD level is the only identified biomarker that predicts treatment failure in *ETV6::RUNX1* leukemia [9, 10]. Although genetic biomarkers have been investigated [25–28], none have yet to been confirmed. To address this gap, we performed a comprehensive genomic analysis of 362 children (median age 4 years, range 1–17 years; 171 females) with newly diagnosed *ETV6::RUNX1*-positive B-ALL treated according to the NOPHO ALL2000 (*n *= 50) or ALL2008 (*n *= 312) protocols (Figs. 1B and S1A-B). The *ETV6::RUNX1* fusion status was clinically verified (See Methods, breakpoints in WGS cohort in Fig. S1C, and additional genomic assay confirmation in Supplementary Table 2). First, we profiled a discovery cohort using WGS (tumor-normal, *n *= 33) and bulk RNA sequencing (RNA-seq; tumor-only, *n *= 51), both data types being available for 31 cases. This was followed by quantitative DNA array CNV (*n *= 141) analysis, and targeted sequencing (*n *= 261) of selected gene regions and genomic backbone to identify SNVs, InDels and CNVs (Figs. 1B and S1A-B; see also Methods, Supplementary Table 1).

In the WGS analyses (*n *= 33), a total of 82,505 somatic SNV (median 1882, range 763–11,046) and 13,990 InDel mutations (median 413, range 153–911) were identified (Fig. S2A). Considering only non-synonymous SNVs and InDels in the coding regions, the median number of variants was 15 (range 8–95) and 6 (range 2–19) per case, respectively (Fig. S2B, Supplementary Table 3), agreeing with previous genomics studies in this subtype [14, 16]. In total, 926 SVs (including deletions < 1 Mb, the most frequent SV type) were identified, with a median of 27 variants per case (range 9–51) (Fig. S2C, Supplementary Table 4). Deletions and gains of > 1 Mb in size were classified as CNVs, and their analysis revealed 156 gains (median 2, range 0–26), with three cases harboring whole-genome duplications, and 153 deletions (median 4, range 0–22). In addition, 19 copy number neutral loss-of-heterozygosity (LOH) events were identified (median 0, range 0–9) (Supplementary Table 5). Across all cases with available CNV data (*n *= 358), 1168 deletions (median 3, range 0-22) and 1015 gains (median 2, range 0–26) were identified (Supplementary Tables 5-7). No significant difference in CNV numbers or fraction of genome affected by copy number changes were observed between responder groups (Fig. S2E-D). Overall, the frequency of somatic genetic changes was not significantly associated with the induction therapy response (Fig. S2A-D), suggesting that genetic diversification may not underlie the drug-resistant phenotype.

### Fast responders display APOBEC mutational signature and features of active cell cycle

**Fig. 2:**
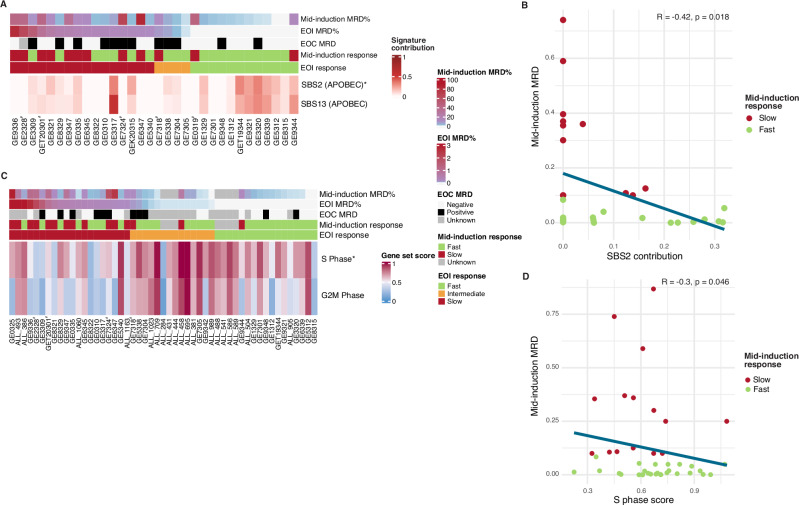
The APOBEC mutational signature and cell cycle activity correlate with a fast treatment response during induction therapy. **A** Mutational signature contributions of APOBEC signatures in the WGS cases (*n *= 33) ordered according to the EOI MRD and visualized as heatmaps. A single asterisk indicates a *p* value < 0.05 (Spearman’s rank correlation coefficient), and significance in mid-induction response group. **B** SBS2 APOBEC mutation signature contribution of the WGS cases (*n *= 33) and its correlation with mid-induction MRD presented as a scatter plot, with Spearman’s correlation coefficient. Red color indicates slow and green fast mid-induction response, respectively. **C** Heatmap visualization of S- and G2M-phase gene set scores by EOI treatment response (RNA-seq cohort, *n *= 51), with a single asterisk indicating *p* value < 0.05 (Spearman’s rank correlation coefficient), and significance in both mid-induction and EOI response groups. **D** S-phase gene set score of the RNA-seq cases (*n *= 51) and its correlation with mid-induction MRD presented as a scatter plot, with Spearman’s correlation coefficient. Red color indicates slow and green fast mid-induction response, respectively. Case IDs with hash symbol indicates relapsed patients in (**A**) and (**C**).

Next, we focused on the sub-cohort with WGS profiles to investigate the underlying mutational mechanisms that may stratify the response groups based on mutational signatures and RAG-mediated recombination events. To this end, we calculated mutational signature scores using somatic SNV profiles from the 33 WGS tumor samples (Supplementary Tables 8–10). Earlier study reported that APOBEC signatures (SBS2 and SBS13) were enriched in *ETV6::RUNX1* leukemia [17]. In our cohort, a moderate correlation of MRD level and the COSMIC signatures corresponding to APOBEC activity was observed based on two different tools (see Methods), with the highest signature scores in patients with a fast early response, as measured by flow cytometry-based MRD at mid-induction (SBS2 *r* = −0.42, *p *< 0.05; SBS13: *r *= −0.35, *p *= 0.05, EOI: SBS2 *r *= −0.33, *p *= 0.06; SBS13 *r *= −0.30, *p *= 0.1, Fig. 2A-B, Fig. S3A-B, Supplementary Tables 8-11). Additionally, positive weak correlations of SBS1 (age, clock-like cell-intrinsic process) (*r *= 0.30, *p *= 0.09) and SBS8 (unknown) (*r *= 0.39, *p *< 0.05) scores with mid-induction MRD levels were observed (Fig. S3A, Supplementary Tables 8, 9, and 11).

The APOBEC mutational signatures originate from the activity of the APOBEC3A (A3A) and APOBEC3B (A3B) cytosine deaminases [10, 29]. Overexpression of the *A3A* and *A3B* genes correlate with the DNA damage response (DDR) associated genes, and enhanced replication stress [25–28]. In addition, there is evidence that *A3A* and *A3B* expression is regulated in a cell cycle -dependent manner in multiple myeloma and healthy blood cells, with the highest level in the G2M-phase [29–31]. Consistent with the literature, single-cell (sc)RNA-seq profiles of healthy bone marrow B cells revealed expression of *A3B* in cycling hematopoietic stem and progenitor cells (HSPCs), pro-B and pre-B cells (Fig. S3C), supporting the fact that *A3B* expression is regulated during the cell cycle. In scRNA-seq data from five *ETV6::RUNX1* leukemias from our previous study [32], *A3B* expression was the highest in the G2M phase cells compared to other cell cycle phases (Fig. S3D). Interestingly, we noticed a negative correlation between MRD and expression level of *A3B* in S-phase cells (*r *= −0.99 and *p *< 0.001) (Fig. S3D). Accordingly, significantly higher *A3B* levels were found in S-phase cells from the cases with mid-induction MRD < 0.1 (*p *< 0.01) (Fig. S3F). Using the larger bulk RNA-sequencing from the NOPHO cohort (*n *= 51), and cell cycle phase-specific genes, we found further evidence to link cell cycle regulation with MRD level. Specifically, the S-phase gene set score demonstrated a negative correlation with MRD levels at both mid-induction (*r *= −0.30, *p *< 0.05, empirical *p* value = 0.05) and EOI (*r *= −0.28, *p *< 0.05, empirical *p* value = 0.04) (Fig. 2C, D). A weaker trend was observed with the G2/M-phase gene set score (Fig. 2C, Supplementary Table 12).

To examine the role of putative transcriptional regulatory mechanisms, we calculated gene set scores from transcription factor (TF) regulons, as defined by Mehtonen et al. [33]. The gene set scores from the E2F3 and E2F8 regulons, which represent critical TFs controlling the G1/S transition [32, 34], were associated with EOI MRD levels (Fig. S3E, Supplementary Table 12). Previously, Somasundaram et al. showed that also PAX5 could control pro-B cell expansion by regulating *MYC* [35]. Li et al. recently showed that re-introduction of ectopic expression of *PAX5* into the *ETV6::RUNX1* positive REH cells led to the partial blockade of G1 to S transition and a drug-resistant phenotype [36]. Examining the patient genomics profiles collected in our study in context of the previously identified mechanisms linking MYC, PAX5 and B cell proliferation, we found that *MYC* was higher expressed in the S-phase cells of fast responding cases (*p *< 0.01) based on the patient scRNA-seq profiles (Fig. S3F, the modest decrease in *PAX5* was not significant). Consistent with the significant correlation with S-phase score in the bulk RNA-seq cohort, deletions overlapping the *PAX5* locus were significantly (*p *< 0.001) more prevalent in fast-responders (Fig. S3G-I, Supplementary Table 4 and 7). Next, we asked whether these TFs that previous mechanistic studies linked with cell cycle regulation could directly regulate *A3B* expression. We found two candidate regulatory regions within the *A3B* locus with co-localized binding of MYC and PAX5, suggesting that A3B and cell cycle entry regulation are tightly coupled (Fig. S3J).

In contrast, high MRD levels at EOI were associated with increased GATA2 regulon activity (*r *= 0.40, *p *< 0.01), and high MRD at mid-induction and EOI with elevated MAX regulon activity at diagnosis (*r *= 0.35, *p *< 0.05 and *r *= 0.44, *p *< 0.01, respectively) (Fig. S3E, Supplementary Table 13).

Overall, these findings suggest that the biological processes that distinguish slow and fast responding leukemias might correspond to specific mutational signatures and TF activities that associate with distinct phases of the cell cycle.

### Frequent immunoglobulin light chain recombination events in treatment resistant cases

**Fig. 3:**
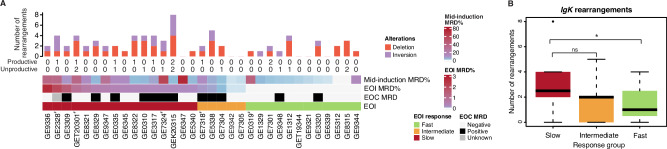
Immunoglobulin light chain rearrangements are enriched in slow responding *ETV6::RUNX1* cells. **A** Bar plot showing number of the *IGK* gene rearrangements by EOI response. Numbers of productive and unproductive rearrangements marked below the bar plot. Case IDs with hash symbol indicates relapsed patients. **B** Number of *IGK* gene rearrangements by EOI response groups as a boxplot. Median value is marked as black vertical line inside of each box. A single asterisk indicates a *p* value < 0.05 (Wilcoxon test). The data in A-B encompass the cases in the WGS cohort (*n *= 33).

Off-targeting activity of RAG enzymes drives the secondary mutational processes in *ETV6::RUNX1* leukemia, causing SVs for example at *TBL1XR1* locus, which is associated with inferior outcome [17]. On the other hand, successful RAG-mediated V(D)J recombination of immunoglobulin (Ig) genes results in strong pro-survival signaling via the pre-B cell receptor (pre-BCR) and B cell receptor (BCR), essential to normal cell functions. This recombination occurs in two stages: initially, the Ig heavy chain (*IGH*) gene is rearranged in pro-B cells, followed by the rearrangement of light chains – first *Ig kappa (IGK)*, then *Ig lambda (IGL)* – in pre-B cells until a productive chain is formed [37–39]. Since leukemic *ETV6::RUNX1* cells resemble pro-B cell stage in both their immunophenotype and transcriptome profiles, it is expected that only Ig heavy chain recombination events are present [33]. However, given the potential plasticity of leukemic blasts, we hypothesized that the active RAG-mediated recombination process might diversify the leukemic Ig repertoire beyond heavy chains. To investigate this, we employed the IgCaller tool [40] on the WGS data and observed that slow responders exhibited more frequently rearrangements in *IGK* genes compared to fast responders (Wilcoxon test: *p *= 0.03) (Fig. 3A-B, Supplementary Tables 14 and 15). Additionally, slow responders harbored several events at the kappa-deletion element, indicating repetitive and frequent recombination attempts within this locus [41, 42] (Fig. S4A-C).

As the increased frequency of Ig light chain rearrangements may provide a survival advantage by enabling active BCR signaling, we performed scRNA-seq coupled with scVDJ-sequencing to analyze the VDJ repertoire in cells collected at diagnosis, day 2, and mid-induction. The case selected for this analysis had a slow-responding leukemia with *IGK*-rearrangement detected in DNA analysis (GEK20315). At diagnosis the bone marrow leukemic blasts exhibited a typical pro-B-like expression profile and expressed only the *IGHM* gene, but not the light chains (Fig. S5A-B). However, light chain expression was highly elevated during the induction therapy as evidenced in the remaining blast-like (*DNTT* + *MME* + ) cells at the mid-induction time point (Fig. S5A-B, non-B cells are shown as controls in Fig. S5C). To verify the blast status of these cells, we compared the most frequent *IGH* rearrangement sequence identified through WGS with the scVDJ-sequencing data and found that cells with the matching heavy chain sequences were exclusively present in the blast-transcriptome matched cells at diagnosis, as well as in the *DNTT* + *MME+* blast cluster observed in day 2 and mid-induction samples (Fig. S6A). Moreover, blasts with productive light chain rearrangement were enriched upon the induction treatment in BM- derived samples from this patient (IGK: from 0.6% to 11%, Fig. S6B, non-B cells are shown as controls in Fig. S6C).

### Presence of deletions in anthracycline- and glucocorticoid-response modulating genes correlate weakly with mid-induction treatment response

Oshima et al. previously conducted an extensive genome-wide CRISPR analysis of chemotherapy-gene interactions in REH cells (*ETV6::RUNX1*-fusion positive cell line derived from a relapsed case) [43]. Their study identified multiple gene knockouts that either enhanced sensitivity or conferred resistance to various chemotherapeutic agents, including vincristine, 6-mercaptopurine, L-asparaginase, cytarabine, methotrexate, daunorubicin, and maphosphamide [42]. To investigate their impact on induction treatment responses, we compared the cell line-based results to our patient-derived data. Since the REH cell line lacks a functional NR3C1 glucocorticoid receptor, we complemented our analysis with data from a dexamethasone screen from the *DUX4*-rearranged (*DUX4::IGH*) Nalm-6 B-ALL cell line [44]. To investigate whether the frequency of CNV deletions present at diagnosis in the screens differs between response groups, we calculated the median number of deletions in different responder groups that impacted the top genes associated with drug resistance or sensitivity if they are inactivated (*n *= 261, Fig. S7). Genes selected for the analysis were identified using several cut-offs; the top 100 and top 200 genes based on resistance/sensitivity ranking, as well as those with an FDR < 0.25. At the CNV level, no significant differences were found in the number of deletions across responder groups for sensitizing or resistance hits from the screens, regardless of the cut-off used (Supplementary Table 16). However, fast responders by mid-induction timepoint were more likely to harbor any daunorubicin- (*p *= 0.017) or dexamethasone-sensitizing deletions compared to slow responders (*p *= 0.058) (Supplementary Table 16). Overall, these findings suggest that a simple stratification based on the CRISPR screen hit deletion frequencies alone could not distinguish suboptimal induction therapy responders in our patient cohort.

### Mutations in transcriptional regulator and tumor suppressor genes associated with treatment response

**Fig. 4:**
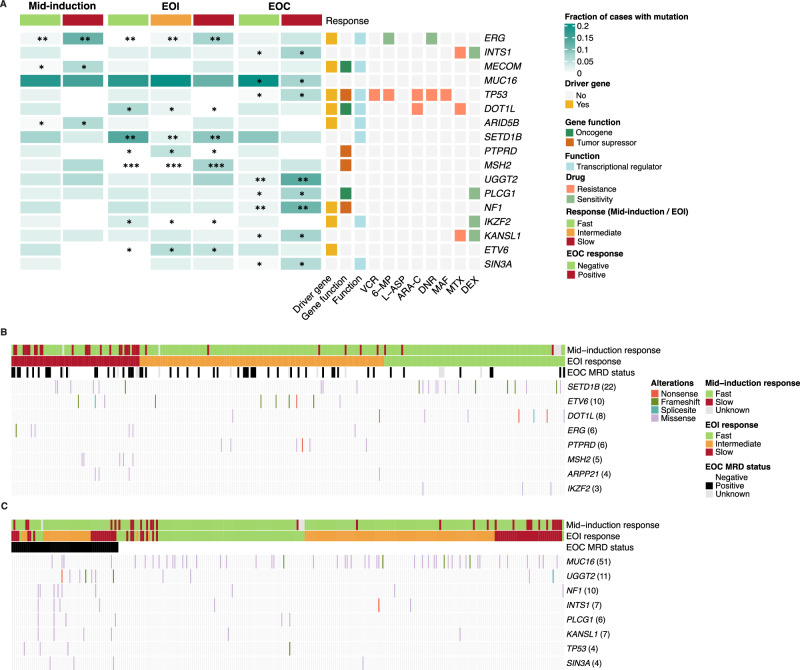
SNVs and InDels associated with treatment response. **A** Heatmap presenting the fraction of cases (total *n *= 294) in each responder group harboring mutations affecting the indicated genes and their effect on drug responses and known biological function. Three asterisks indicate a *p* value < 0.001, two asterisks indicate a *p* value < 0.01, and a single asterisk indicates a *p* value < 0.05 in Fisher’s exact test. The drug resistance/sensitivity annotations are based on the drug screen results by Oshima et al. [43] and Poulard et al. [44]. **B**,** C** Oncoprints visualizing gene mutations significantly associated with (**B**) EOI response and (**C**) EOC MRD status (*n *= 294). Numbers of the mutations are indicated in parentheses. EOI end of induction, EOC end of consolidation, 6-MP 6-mercaptopurine, ARA-C cytarabine, DNR daunorubicin, L-ASP L-Asparaginase, MAF maphosphamide, MTX methotrexate, VCR vincristine.

To further examine whether the genes associated with the treatment response [43, 44] harbored missense or potentially deleterious SNVs and InDels, we designed a gene panel where we included candidate response-modulating genes identified through our WGS discovery cohort and the cell line CRISPR screens. We combined variant analysis from WGS and panel sequencing of diagnostic samples (total patients *n *= 294, Supplementary Table 3, Supplementary Tables 17–19), focusing on SNVs and InDels with predicted impact on protein function. Panel-of-normals (PON)- and population-based allele frequency (AF) filtering was applied to remove the common germline variants, however it is noteworthy that germline mutations may be present at low frequencies. Variants that associated with treatment response (Fisher’s exact test, Supplementary Table 18) affected genes functioning in transcriptional regulation, like *ARID5B*, *SIN3A*, *INTS1*, and *KANSL1*, and suppression of oncogenes, notably including the driver genes *TP53*, *ETV6* and *NF1* [45] and seven genes identified from the CRISPR-based drug sensitivity or resistance screen, such as *IKZF2* [43, 44] (Fig. 4A-C).

The transcriptional regulator gene *ARID5B*, which was more frequently mutated among the slow responders to induction therapy (proportion of slow vs. fast responders carrying a mutation: 0.059 vs. 0.01 based on mid-induction MRD, *p *< 0.05; 0.029 vs. 0.01 based on EOI MRD *p *< 0.1), consistent with previous reports linking it to poor treatment outcome in ALL [46]. The tumor suppressor gene *MSH2* [47], was mutated among EOI slow responders (proportion of slow responders carrying a mutation 0.072, EOI MRD, *p *< 0.05), while no mutations were found within the fast or intermediate groups. In addition, mutations in *ETV6* were more common in slow and intermediate responders (EOI MRD, *p *< 0.05). In comparison, we observed a higher frequency of *IKZF2* mutations among fast responders, in agreement with previous reports linking these mutations to dexamethasone sensitivity (proportion of slow vs. fast responders carrying a mutation: 0.031 vs. 0.000, EOI MRD *p *< 0.0.5) (Fig. 4A, Supplementary Tables 18 and 19).

Many mutations identified in our analysis associated with EOC response. The tumor suppressor gene *NF1*, which regulates the Ras pathway [48], exhibited mutations significantly more frequently in patients with positive EOC MRD (proportion of MRD positive vs. negative patients carrying a mutation: 0.111 vs. 0.013, EOC MRD *p *< 0.01). Similarly, these patients showed a significantly higher frequency of mutations in *UGGT2* (proportion of MRD positive vs. negative patients carrying a mutation: 0.130 vs. 0.018, EOC MRD *p *< 0.01), and the transcriptional regulator *SIN3A* (proportion of MRD positive vs. negative patients carrying a mutation: 0.056 vs. 0.004, EOC MRD *p *< 0.05). *TP53* mutations (proportion of MRD positive vs. negative patients carrying a mutation: 0.056 vs. 0.004, EOC MRD *p *< 0.05), which are linked to multidrug resistance [43, 47, 49], and methotrexate resistance -associated mutations in *KANSL1* (proportion of MRD positive vs. negative patients carrying a mutation: 0.074 vs. 0.009, EOC MRD *p *< 0.05) and *INTS1* (proportion of MRD positive vs. negative patients carrying a mutation: 0.074 vs. 0.013, EOC MRD *p *< 0.05) genes were significantly more frequent in patients with positive MRD at the EOC time-point [43]. The latter associations may bear clinical significance as patients had received high-dose methotrexate treatments by that time-point, suggesting a possible mechanism of resistance.

### Chromosomes 3, 6, 9, 12, 15 and 21 harbor gene loci that associate with treatment response at copy number level

**Fig. 5:**
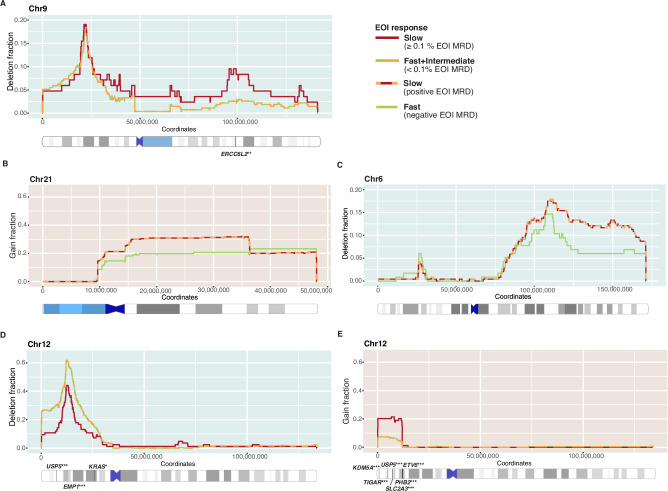
CNV alterations associating with treatment response status at EOI. Fraction of cases in each indicated EOI response group with (**A**) deletion at chromosome 9, (**B**) gain at chromosome 21, (**C**) deletion at chromosome 6, (**D**) partial deletion or (**E**) gain at chromosome 12. Red curves represent the fraction of slow responders (EOI MRD ≥ 0.1%, *n *= 84) harboring the indicated copy number change at each region of the chromosome, whereas the dashed green-orange curves indicate the same for fast and intermediate responders (EOI MRD < 0.1%, *n *= 272). Dashed red-orange curves represent the fraction of slow and intermediate responders (EOI MRD > 0%, *n *= 240) harboring the indicated copy number change at each region of the chromosome, whereas the green curves indicate the same for fast responders (EOI MRD = 0%, *n *= 116). The chromosome structure with relevant expressed genes and their empirical *p* values visualized below the curves. Grey bars indicate cytobands and black lines indicate the location of highlighted genes. Three asterisks indicate an empirical *p* value < 0.001, two asterisks indicate an empirical *p* value < 0.01, and a single asterisk indicates an empirical *p* value < 0.05.

*ETV6::RUNX1* genomes typically contain a few large alterations, such as deletions in the non-rearranged chromosome 12 and gains of the der(21)t(12;21) chromosome. To identify response stratifying copy number changes (gains and deletions > 1 Mb in size), we combined the WGS (*n *= 33), DNA array (*n *= 70) and panel sequencing (*n *= 255) CNV data (in total *n *= 358). The CNV locations (within chromosome arm) and response groups labels were randomized to calculate empirical *p* values and to assess the association between therapy response and each gene region with detectable RNA expression based on the RNA-seq cohort (Supplementary Tables 20-21). Deletions in the long arm of chromosome 9 were more common in mid-induction and EOI slow responders, affecting the genes such as *SYK*, *INIP* and *ERCC6L2* (empirical *p *< 0.01, Figs. 5A and S8A). The partial gain of chromosome 21, corresponding to the der(21)t(12;21) gain (empirical *p *< 0.05, Fig. 5B), and the partial deletion of the chromosome 6q were associated with slower EOI response (empirical *p *< 0.05, Fig. 5C). Furthermore, CNVs associated with EOC MRD positivity included focal deletions on chromosome 3 (centered at the *TBL1XR1* gene locus, *p *< 0.001, Fig. S8B), and on the chromosome 15 (*INO80* locus, empirical *p *< 0.001, Fig. S8C). Presence of *TBL1XR1* alterations agrees with the recent finding by Brady et al. [17] which associated the locus with inferior outcome in *ETV6::RUNX1* leukemia [17].

In fast-responding cases at all studied timepoints, we found more frequent deletions of chromosome 12p (empirical *p *< 0.05, Figs. 5D and S8D and E). This region that includes the *ETV6* locus typically encompassed a larger segment beyond the translocation breakpoints, notably including the well-known oncogene *KRAS*, and the *EMP1* gene locus, whose deletion has been shown to sensitize cells to prednisolone and high expression associated with poor event-free survival (EFS) in B-ALL [50, 51]. In contrast, slow-responding patients exhibited more frequent gains at 12p, covering a shorter region, approximately 12 Mb from the start of chromosome 12, corresponding to the der(21)t(12;21) gain (Fig. 5E). The overlapping region encompassed expressed genes including *ETV6*, *TIGAR*, *SLC2A3, KDM5A, PHB2*, and *USP5* with significant gains in slow responder group across all time points (empirical *p *< 0.05, Figs. 5E and S8F-G). Several of these genes have been previously associated with chemoresistance and tumorigenesis [52–60].

### Multiple gene loci within chr12 underlie differential drug responses ex vivo and in vitro

**Fig. 6:**
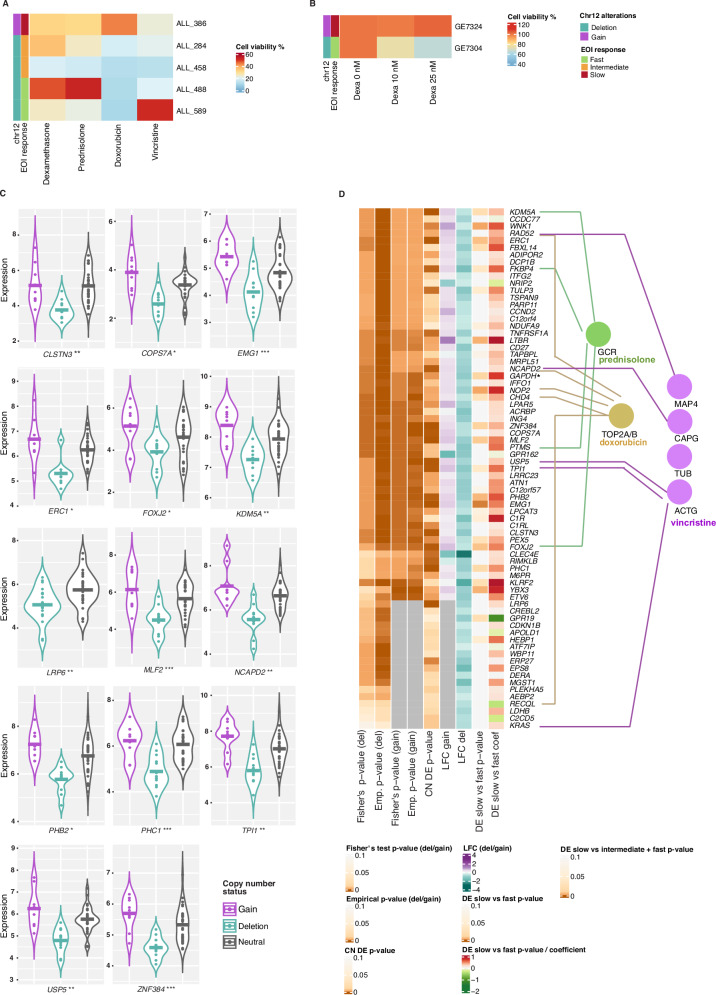
Ex vivo drug responses. **A** Ex vivo drug response of cells from five *ETV6::RUNX1* patients harboring a CNV at chromosome 12. CNV status, EOI response groups and drug responses to drugs used in induction therapy are shown (concentrations: dexamethasone 1.4 µg/ml (3567 nM), doxorubicine 0.5 µg/ml (920 nM), prednisolone 50 µg/ml (138 uM), and vincristine 2.5 µg/ml (3000 nM)). **B** Ex vivo dexamethasone response of two patients harboring CNV alteration at chromosome 12. CNV status and EOI response group are shown as in (**A**). **C** Violin plots showing the copy number effect on gene expression of selected genes in chromosome 12. The expression is reported for cases in the RNA-seq cohort (*n *= 50) and the values are CPM-normalized and log2-transformed. The horizontal line indicates the mean expression level in each group. Three asterisks indicate FDR < 0.01, two asterisks indicate FDR < 0.05, and a single asterisk indicates FDR < 0.1. **D** Summary of genes affected at expression level by chromosome 12 CNV. Statistical associations in genomics analyses, and protein-level interactions with drug target proteins are shown (prednisolone in green, doxorubicin in brown and vincristine in purple). The genes shown are expressed in leukemic cells and significant in the EOI Fisher’s exact tests comparing deletion/gain prevalence (*n *= 356) and the gene expression copy number DE analysis at EOI treatment groups (slow vs. intermediate + fast) (*n *= 50, *p *< 0.05). Genes are ordered according to their chr location (chromosome start at the top), and the heatmap visualizes the statistical test *p* value or log fold change, as indicated. DE differential expression, del deletion, Emp *p* value empirical *p* value, EOI end of induction, EOC end of consolidation, LFC log fold change.

We chose the chr12 p-arm alterations for further analysis based on the opposing pattern of gains versus deletions in slow- and fast-responders, respectively. To further assess their combined impact on drug responses, we analyzed ex vivo drug responses (Fig. 6A, B). Four patients with a 12p deletion exhibited sensitivity to induction therapy drugs (dexamethasone, doxorubicin, prednisolone, or vincristine), whereas one patient with a 12p gain demonstrated resistance to these drugs. Additionally, in a dexamethasone-only screen, one patient with a 12p deletion showed sensitivity across the tested dexamethasone concentrations, whereas the patient with a 12p gain was resistant to dexamethasone, in agreement with the MRD associations.

Previous studies have shown that the activating *KRAS* G13 mutation confers resistance to prednisolone, methotrexate and vincristine, while the *KRAS* G12 mutation shows conflicting effects on drug responses [61, 62]. In our analysis, *KRAS* was more frequently deleted in fast responders at EOI (chr12p, Fig. 5D) and higher expressed in cases with positive EOC MRD (EOC MRD, *p *= 0.019, Supplementary Table 22). For comparison, we chose the *NT5DC1* gene, which was among the top genes whose loss sensitized cells to methotrexate in the CRISPR screen [43], and carried SV deletions in two patients with slow treatment response in our cohort. We deleted both gene separately in REH cells (Fig. S10A-C), and assessed sensitivity to both vincristine and methotrexate. KRAS knockout (KO) increased sensitivity to both drugs (*p *< 0.01, Fig. S10D-E), while NT5DC1 KO specifically enhanced methotrexate sensitivity (*p *< 0.05) compared to REH wild-type (WT) cells in vitro (Fig. S10F).

Overall, out of 377 genes expressed in BM samples and located in response-associated CNV regions, the expression of 178 genes was significantly influenced by their copy number (CN) status (*p *< 0.05) (Fig. S11, Supplementary Tables 20-21). The levels for top markers for chromosome 12p, *CLSTN3*, *COPS7A*, *EMG1*, *ERC1*, *FOXJ2*, *KDM5A*, *LRP6*, *MLF2*, *NCAPD2*, *PHB2*, *PHC1*, *TPI1*, *USP5*, and *ZNF384 (p *< 0.01, FDR < 0.1), are shown as violin plots in Fig. 6C. Generally, changes in CN led to the corresponding alterations in gene expression. However, considering that additional epigenetic mechanisms can render a similar functional effect, we performed an additional MRD-stratified response analysis based on the RNA-seq data, focusing on the genes residing within the significant CNV regions (Supplementary Table 22). Further integrating the scRNA-seq data, we could distinguish 73 genes from chr 12 that were expressed in leukemic cells and significantly correlated with CN status at bulk RNA-seq level (Fig. 6D, see also Fig. S11 for other CNV regions). These included 14 genes encoding proteins that directly interact with the target proteins for the induction therapy drugs prednisolone, doxorubicin and vincristine (Fig. 6D), including FKBP4 that regulates glucocorticoid receptor nuclear transport upon ligand binding and NCAPD2 that represents a subunit of the condensin multiprotein complex required for mitotic chromosome condensation with previously reported synthetic lethal interaction with KRAS [63]. In total, 33/73 genes showed a loss of fitness phenotype in cell line screens representing hematopoietic and lymphoid tissue (Supplementary Table 24). Moreover, the gene-level interaction network (Fig. S10G-I), shortest paths from genes to drug target genes through shared biological process, molecular function ontology terms and perturbation assay nodes based on the knowledge graph SPOKE showed significant difference to random gene sets of the same size based on multiple network metrics (Fig. S10I).

Our integrative analysis highlights the functional relevance of chromosome 12p alterations in treatment response, linking CNV-driven gene expression changes to drug sensitivity. The association of KRAS loss with increased vincristine and methotrexate sensitivity in vitro, along with the identification of genes directly interacting with induction therapy targets, underscores the biological impact of these alterations. These findings provide a foundation for refining response prediction and developing more personalized treatment strategies in leukemia.

## Discussion

Recent studies have revealed novel genetic alterations that define subtypes in ALL and have linked these alterations to patient outcomes. Nevertheless, the absence of consistent diagnostic genetic biomarkers for predicting treatment response remains an unmet need, which served as the motivation for this study. We focused on the *ETV6::RUNX1* subtype of ALL and identified genomic alterations present at diagnosis that associated with early treatment response, which is a strong predictor of relapse risk. Although the overall mutation rate did not stratify risk groups, the genomic signatures indicated that distinct mutational processes related to RAG and APOBEC activities may shape the genetic landscapes of slow versus fast responders, respectively. Moreover, the MRD risk groups differed in gains versus deletions of chromosome 12p that harbored multiple genes affecting the induction therapy drug response pathways. Additionally, we identified five other large CNV alterations and mutations in specific transcriptional regulator and tumor suppressor genes that were associated with chemotherapy response. Overall, our findings highlight SNV, CNV, and transcriptomic alterations impacting the induction and consolidation treatment response among patients with *ETV6::RUNX1* leukemia paving the way for more tailored therapy allocation.

Mutational signatures are a valuable tool for elucidating the mutagenic processes that drive cancer development and have prognostic significance in patients [64]. In breast, lung, and bladder cancer APOBEC3 levels correlate with APOBEC3-induced mutation burden and are associated with clinical outcomes and treatment responses [65–67]. Consistent with previous studies in ALL, our findings showed that the APOBEC mutational signatures (C → T and C → G conversions) are prevalent in *ETV6::RUNX1* leukemia [14, 17]. Notably, our study uncovered that this signature is particularly enriched in patients who exhibited an early fast treatment response measured by mid-induction MRD. Moreover, we showed that *A3B* S-phase expression at single cell level and the gene set scores for S-phase in bulk RNA-seq negatively correlated with MRD. Previous pan-cancer analyses have demonstrated that *A3B* expression correlates with cell cycle and DNA repair genes, unlike other family members that play a role in immune defense. *A3B* overexpression can therefore contribute to tumorigenesis by driving replication stress and chromosomal instability [27, 31, 67–69]. We demonstrated that cycling precursor B and *ETV6::RUNX1* leukemic cells had elevated levels of *A3B* mRNA and associated its expression with the regulation of G1/S-transition. The *APOBEC3B* locus harbored binding sites for the regulatory circuit for pro-B cell expansion involving PAX5 and MYC. Our results on *A3B* expression in actively cycling leukemic blast are consistent with the previously proposed tumorigenic mechanism and provide a compelling rationale for further investigations into the interplay between cell cycle regulation, APOBEC mutational signatures, and therapy response in B-ALL.

Previous WGS analysis of *ETV6::RUNX1* leukemias established a key role for the RAG-driven mutational process in the generation of SV at off-target sites [14]. Surprisingly, in our analysis slow responders exhibited more frequent Ig-kappa rearrangements and kappa-deleting elements than fast responders. We have earlier demonstrated that although diagnostic *ETV6::RUNX1* blast cells closely resemble healthy cells at the pro-B cell stage, cells collected at mid-induction therapy shifted to a pre-B cell-like phenotype [32]. Moreover, B-ALL cells which were more resistant to glucocorticoids were reported to exhibit a pre-B cell-like phenotype [70], which makes it plausible that light gene region rearrangements typical of pre-B cell stage could become targets for recombination. Single-cell VDJ profiles during induction chemotherapy of a slow-responding case studied here confirmed that blast cells expressed immunoglobulin light chains, with productive rearrangements enriched in residual blasts after two weeks of induction therapy. Previously, Kansal et al. (2014) published a report featuring nine diagnostic pediatric precursor B-ALLs with dim IGK expression, including one *ETV6::RUNX1* patient [71]. In Ph+ ALL, light chain rearrangements are known to promote the expression of *BCL6*, a protein associated with survival and drug resistance [72–74], whereas in B cell lymphomas, active BCR signaling has been shown to drive tumorigenesis and support leukemia cell survival [73, 75]. Whether *ETV6::RUNX1* blast cells with productive rearrangements acquire a survival advantage through active BCR signaling should be explored in future studies at protein level. In addition, the role of enriched deletions in chromosome 9 spanning *ERCC6L2* that functions in double-strand break end-joining in V(D)J merits further investigation [76]. Alternatively, drug-resistant cells could rely on GATA2 and MYC/MAX activities implicated by the positive correlation of MRD with the respective regulon activity scores. *GATA2*, whose expression is typically elevated in hematopoietic progenitor cells, is also highly expressed in *ETV6::RUNX1* samples at both diagnosis and remission [33]. The two alternative phenotypes were found in *KMT2A*-rearranged cells, in which *RUNX1*, *GATA2* and *MYC* down-regulation led to the emergence of drug tolerant cells with active pre-BCR signaling [77].

Overall, our analysis implicated the significant contribution of transcriptional regulators to the differential treatment response. Mutations in the TFs *ERG* and *ETV6* and the histone demethylase complex subunit *ARID5B*, previously linked to poor treatment outcomes in ALL [46, 78], were prevalent in EOI slow responders, while mutations in *IKZF2* and the histone H3K79 methyltransferase *DOT1L* were enriched in fast responders. The impact of *ETV6* deletions on clinical outcomes remains contentious, with some studies indicating a favorable prognosis [79, 80], while others reporting neutral or adverse outcomes [81, 82]. Chang et al. [80] recently reported a lower EFS probability in *ETV6::RUNX1* patients with unbalanced chromosome 21 translocations compared to those with balanced events [80]. We observed that the gain of der(21)t(12;21), previously associated with poor outcome and relapsed disease [83, 84], was more common in slow responders, while deletion of 12p was more frequent in fast responders. Combined, these results suggest that the type of alteration and dosage of the *ETV6* gene are linked to drug sensitivity, with low dosage associated with fast response and high dosage or a mutated gene with slow response. At CNV level, the chr12 alterations further impacted the expression level of the TFs *ZNF384* and *FOXJ2*, representing previously identified fusion partners in B-ALL [85], the RNA binding factor *YBX3* previously linked to pre-B cell expansion [86], the chromatin remodeling complex subunit *CHD4* reported to control lineage-switching and drug resistance in leukemia [87, 88], the lysine demethylase *KDM5A* linked to oncogenic functions and drug tolerance [58, 89], and two Polycomb repressive complex subunits, *AEBP2* and *PHC1* involved in B cell maturation and ALL [90].

Unexpectedly, when we compared top CNV hits associated with drug-sensitivity from in vitro genetic screen in *ETV6::RUNX1*-fusion positive REH cells with patient-derived data, we only found a weak association between daunorubicin- or dexamethasone-sensitizing deletions and mid-induction response. This underscores the importance of validating potentially clinically relevant in vitro findings in patient data, as cell lines often have heavily altered genomes and may better model relapsed disease [91]. From the known oncogenic pathways, the strongest link in our analysis was found to the RAS pathway. In pediatric B-ALL, RAS pathway mutations have been linked to relapse and chemotherapy resistance, including prednisolone resistance, which can be overcome with RAS pathway inhibition [61]. *RAS* mutations occur in about 15% of B-ALL cases; however, their prognostic impact is not well studied. Notably, almost all previous research has focused on activating mutations of *KRAS* or *NRAS* [61]. Our genome-wide analysis provides new information on the role of RAS pathway alterations that relate to treatment response. Deleterious mutations of *NF1*, a well-known negative regulator of the RAS pathway, were more common among patients with residual disease at EOC. Loss-of-function mutations in this tumor suppressor result in the accumulation of active, GTP-bound form of RAS and associates with juvenile myelomonocytic leukemia and AML, but rarely ALL [48, 92]. Similarly, mutations of *PLCG1* were more common among the patients having positive MRD at EOC. Activation of PLCG1 is directly or indirectly related to many signaling pathways, including RAS and BCR [93]. Further studies are needed to explore whether mutations that we identified in *PLCG1* exhibit gain-of-function properties and thereby contribute to treatment resistance by activating the Ras pathway. Finally, chromosome 12 deletions that impacted also *KRAS* were more frequent in fast responders. This was consistent with our KRAS KO model, where KO cells showed enhanced sensitivity to methotrexate and vincristine, consistent with direct protein-protein interaction of KRAS with the drug target proteins.

Previous research has shown that certain mutations are chemotherapy-induced, particularly by thiopurines such as 6-mercaptopurine, and can contribute to relapse in ALL [47]. Notably, *MSH2* mutations have been detected in over 50% of relapsed ALL cases but are generally undetectable at diagnosis [47]. However, our in-depth targeted sequencing revealed that while *MSH2* mutations were rare, they were already present at diagnosis and associated with a slow EOI treatment response. Similarly, our finding that *TP53* mutations, alongside RAS pathway mutations, were present already at diagnosis in patients with measurable disease at EOC, suggests that patients at risk of therapy resistance could be detected early. Mutations in *INTS1*, a component of the Integrator complex involved in small nuclear RNA processing, and *KANSL1*, a subunit of the NSL histone acetyltransferase complex, have previously been associated with methotrexate resistance [45], while *PLCG1* encodes an enzyme that catalyzes the formation of IP3 and DAG from phosphatidylinositol 4,5-bisphosphate, contributing to cell growth, apoptosis, and proliferation [93]. We found two cases carrying these mutations. These mutations are rare and were not found together in pediatric ALL studies but show tendency to co-occur in large pan-cancer studies [94–96]. Since these mutations are not found in other genomic studies related to *ETV6::RUNX1*, it is possible that these treatment response-related mutations are of germline origin, as filtering using a matched normal sample was not applicable.

Cohort size represents a limitation in studies focused on a specific subtype. Here, we obtained available diagnostic DNA samples from *ETV6::RUNX1* cases treated under two successive NOPHO protocols. We leveraged the detailed therapy response data, which was integrated into patient stratification in the latest NOPHO ALL2008 protocol, from which the majority of cases were derived. In future, the ongoing ALLTogether trial in Europe, which tracks MRD levels and uses EOC in addition to EOI in standard assessment, could help validate the prognostic DNA and RNA signatures identified. One limitation of our study is that each biomarker was assessed separately. In future, training classifier models that test the predictive power of multiple response-modifying alterations in combination and identify an optimal subset of predictive features is warranted. Moreover, due to differences in diagnostic practices, some of our findings may be more readily translatable for clinical application in prospective trials. SNP or comparative genomic hybridization (CGH) array data, which are routinely analyzed within many treatment protocols enables the detection of numerous SVs and CNVs approximately with a resolution of 50–200 kb. For instance, they can reliably identify 12p alterations, der(21) amplifications, and other alterations in clinical settings, including the deletions affecting the *TBL1XR1* or *INO80* genes in chromosome 3 and 15, respectively. Additionally, all variants (within the assay resolution) deviating from normal variation are reported, making this type of analysis a straightforward addition to current risk stratification protocols.

Furthermore, targeted analysis of specific gene loci using panel sequencing may also serve as clinically feasible approach to monitor relevant biomarkers. As demonstrated by our approach, identification of larger CNVs (> 1 Mb) genome-wide is feasible from both methylation array and targeted panel sequencing data if the panel design contains sufficient backbone probes throughout the genome. Identification of more focal structural variants requires higher probe density, and therefore the CNV results in the current study focus on the genome-wide events > 1 Mb in size, although we acknowledge that more focal events could also have relevance in therapy response. However, if a gene region is specifically targeted, like *PAX5* in our panel design, identification of more focal events would be possible also in the clinical context. For instance, many clinical laboratories routinely use tumor gene panels, primarily for solid tumors, which almost invariably include the *KRAS* gene, enabling the incorporation of detection of individual gene alterations also from the *ETV6::RUNX1* ALL samples to diagnostic workflow. In contrast, the clinical implementation of mutational signatures and RNA-seq –based findings, such as those related to cell cycle activity, remains more challenging. Mutational signature analysis requires deep genome-wide sequencing, which is not routinely performed in most clinical settings. Likewise, the limited use of RNA sequencing in routine diagnostics continues to constrain the clinical applicability of these potential biomarkers.

While this work was in revision, Li et al. [36] extended the characterization of *ETV6::RUNX1* patients with RNA-level analyses and mutational data from three cohorts, where *ETV6::RUNX1* patients were divided into two groups based on the transcriptomic profiles [36]. Their analysis found higher expression of *GATA2* in slow responders, APOBEC mutation signatures and more *PAX5* deletions in the patient cluster with fast early treatment response, in agreement with our findings. However, chromosome 21 gains that were detected based on RNA-seq copy number analyses associated in this cohort with the fast-responder group. At mechanistic level, their findings that the reintroduction of *PAX5* into REH cells induced cell cycle arrest and resulted in a more drug-resistant phenotype [36] were consistent with previous mouse study by Somasundaram et al. [35] that explored the MYC/PAX5 axis in pro-B cell development and B-cell acute lymphoblastic leukemia. Together, the patient data in our study and Li et al. [35] suggest that in the leukemic context, cells with reduced PAX5 may display a more proliferative phenotype that distinguishes fast and slow responders *ETV6::RUNX1* cases. However, neither study addressed whether PAX5 deletion status (homozygous or heterozygous) should be distinguished in this context. This would be important to confirm in future mechanistic studies.

In summary, we integrated multiomic data with treatment response profiles to characterize the genetic alterations and biological processes associated with therapy response in pediatric *ETV6::RUNX1* leukemia. Our findings highlight that APOBEC- and RAG-driven mutational processes distinguish fast and slow responders, respectively, while also providing new insights into the significance of reciprocal chromosome 12 alterations, and its impact to treatment response. The identified DNA and RNA markers, which correlate with MRD levels at both EOI and EOC, pave the way for improved disease classification at diagnosis.

## Material and methods

Detailed Materials and Methods in Supplementary material.

### Patient cohort and response groups

Samples from 365 *ETV6::RUNX1* ALL patients in total were analyzed by WGS, methylation array, RNA-seq and/or targeted panel sequencing (Supplementary Table 1). The *ETV6::RUNX1* fusion status was identified clinically from all patients (Supplementary Table 2). Additionally, the t(12;21) resulting in the *ETV6::RUNX1* fusion gene was detected in 32/33 cases analyzed by WGS. For the remaining case (GE7324), the fusion was detected in the clinics by conventional fusion-PCR method, but a more complex event was detected by WGS, as the *ETV6::RUNX1* fusion was generated through a combined deletion and translocation event involving the neighboring gene locus. In addition, *ETV6::RUNX1* fusions were detected for 18 cases from RNA-sequencing data using FusionCatcher, and machine learning (ML) model ALLIUM [97] assigned 166/175 to the *ETV6::RUNX1* subtype based on gene expression and/or methylation data (Supplementary Table 2).

The patients were treated according to NOPHO ALL2000 and NOPHO ALL2008 protocols [98, 99], which consisted of induction, consolidation, intensification, and maintenance phases [98, 99]. Four-week induction therapy included glucocorticosteroids, vincristine, doxorubicin, and intrathecal methotrexate. Bone marrow or blood samples were collected before the initiation of therapy and at remission, additional blood samples was collected at day 2 after induction therapy, and at mid-induction. Treatment response measured by MRD was determined by bone marrow sampling at mid-induction, EOI, and EOC using morphology, flow cytometry, and/or ASO-PCR (Fig. 1B, Supplementary Table 2). The responder groups of this study were determined as follows: mid-induction slow group with MRD 10% and fast group with MRD < 10% at day 15, EOI slow group with MRD 0.1%, intermediate group with MRD < 0.1% & > 0%, and fast group with MRD = 0 at day 29, EOC slow group with MRD > 0% and fast group with MRD = 0% at day 79 of treatment.

### Library preparation and sequencing

Library preparation and WGS was performed in five different batches (See Supplementary Materials and Methods). Paired-end sequencing (150 bp) was performed for all sample libraries: diagnostic samples were sequenced at the depth of 60× or 90× and remission samples at 30×. The paired-end RNA libraries were prepared and sequenced in four different batches. Library preparation for targeted panel sequencing libraries of tumor samples (*n *= 261), and the remission samples for the panel-of-normals (PON, *n *= 38) is described in Supplementary Materials and Methods.

### Sequencing data analysis and variant calling

Both WGS (*n *= 33, tumor and normal samples) and targeted panel sequencing (261 tumor samples and PON of 38 samples) data were analyzed by using the BALSAMIC workflow v8.2.10 and v16.0.0 (https://balsamic.readthedocs.io/en/v8.2.10/, https://balsamic.readthedocs.io/en/latest/), respectively, utilizing the tumor-normal mode in WGS and tumor-only mode in panel sequencing data analysis. The pipeline performed read alignment to GRCh37 (utilizing Sentieon for WGS and BWA-MEM for panel sequencing data), produced quality metrics, and conducted variant calling (see Supplementary Materials and Methods). From the WGS data, SNVs and InDels were identified by TNscope, SVs by Manta and Delly, and CNVs by AscatNGS. From the panel sequencing data, SNVs and InDels were identified by VarDict and CNVs by CNVkit, in which analysis the  PON was used to normalize the log2 values The variants were further filtered based on several parameters and quality metrics, including read evidence and size, and variants overlapping with Encode Blacklist regions [100] were excluded (see supplementary Materials and Methods).

VEP and AnnotSV were employed in the annotation of SNVs/InDels and SVs/CNVs, respectively, and coding variants were determined based on the annotations (see supplementary Materials and Methods). Moreover, methylation array data (*n *= 144) [101, 102] was analyzed by utilizing the methylumi [103] and conumee R [104] packages to yield the copy number profiles of the patients. Similarly to WGS data, the CNVs identified from methylation array and panel sequencing data were filtered based on size and overlap with Encode Blacklist regions. In addition, mutational signature analysis was performed using MuSiCa [105], and SigProfilerAssignment [106] using the filtered SNVs (all variants, coding and non-coding) from WGS as input. Musica analysis was made using the online tool provided by the authors using the default settings (https://github.com/marcos-diazg/musica) and version 2 COSMIC signatures and vcf-files as input. SigProfilerAssignment analysis was respectively made using the online tool provided by the authors (https://github.com/AlexandrovLab/SigProfilerAssignment/tree/main/SigProfilerAssignment, https://cancer.sanger.ac.uk/signatures/assignment/app/) with the default settings using the version 3.4 COSMIC signatures and vcf-files as an input. The aligned WGS data was also used as input for the IgCaller tool [40], which identified the immunoglobulin gene rearrangements present in the tumor samples. The rearrangement functionalities (productive/unproductive) were estimated by IgCaller, and complemented with IMGT/V-QUEST and IgBlast annotations.

### Gene expression data analysis

RNA-seq data was available from 51 diagnostic patient samples, and the raw data was processed by using the nf-core/rnaseq workflow [107], which trimmed the paired-end reads, performed read alignment to GRCh38 with STAR, and quantified transcript abundance with the Salmon tool, which was summarized to gene level by tximeta R package. The EdgeR(64) and limma R [108] packages were utilized in data normalization and differential expression (DE) analyses, respectively. The DE analyses encompassed all the response group comparisons (mid-induction, EOI, EOC, *n *= 51) and copy number status comparisons (*n *= 50) for 377 genes arising from the CNV analysis. In addition, the expression of S and G2/M phase -associated genes was assessed, and gene set scores calculated for related regulons. See Supplementary Materials and Methods for details.

### Determination of response group-differentiating gene alterations

To distinguish mutations and CNVs that differentiate the responder groups, the prevalence of recurrent SNVs and InDels (WGS and panel sequencing, *n *= 294) and CNVs (WGS, panel sequencing and methylation array, *n *= 358) among the different responder groups were compared by utilizing the Fisher’s exact test. The top hits from the SNV/InDel analysis were further annotated with functional consequences using the cBioPortal MutationMapper tool to yield a list of variants more likely to impact the encoded protein. The Fisher’s exact tests were repeated for these functionally filtered variants, and results compared to drug response -associated genes from CRISPR screens conducted in ALL cell lines, REH [43] and NALM6 [44] (Fig. S9B, Supplementary Materials and Methods). The top genes affected by CNVs distinguishing the response groups, in turn, were further filtered based on the available gene expression data (*n *= 50), excluding genes lowly expressed across all responder groups or with expression evidence conflicting with the copy number status, 377 genes of interest remaining. To assess if the response group differences at these gene loci would be likely to arise randomly, empirical *p* values were determined by randomizing the CNV coordinates and response groups and comparing the results to patient data (Fig. S9A, Supplementary Materials and Methods). Moreover, the CNV deletions were also compared to the top CRISPR screen genes associated to sensitivity/resistance to ALL drugs [43, 44] to assess if the prevalence of deletions affecting these genes differs among the responder groups. To this end, empirical *p* values were also determined to test if the response group differences are likely to arise only regarding the drug response -associated genes or any random set of genes.

### Single cell genomics from primary ALL cells

Bone marrow or blood mononuclear cells collected at diagnosis, at day 2 and at mid-induction (day 15) were analyzed from cryopreserved samples. Libraries were prepared for transcriptome, cell surface protein marker (ADT) and BCR VDJ-rearrangement analysis, see detailed description from Supplementary Materials and Methods.

### In vitro and ex vivo drug screens

In vitro methotrexate and vincristine responses were studied using CRISPR-Cas9 engineered REH KRAS and NT5DC1 knockout cells (see Supplementary Materials and Methods). Ex vivo drug screens were performed using diagnostic bone marrow samples [109] (see Supplementary Materials and Methods).

### Statistics

The cumulative incidence (CI) of relapse and death were calculated by using the Fine-Gray model, where death in CR was treated as a competing event. Correlations between COSMIC mutational signatures, gene set expression and TF regulons to response measured by MRD were calculated using Spearman’s rank correlation test. Fisher’s exact tests were used to study the top response group -differentiating gene hits (SNVs and InDels, CNVs). The significance threshold for DNA alterations was set at 0.1 based on power calculations carried out for MRD response group sizes and different variant prevalence levels. To complement this analysis, empirical *p* values for the response-group differentiating CNVs were determined as described in section titled “Determination of response-group differentiating gene alterations”. Wilcoxon test was used to calculate the difference in number of *IGK* rearrangements between slow and fast (combined with intermediate group) responders. In the RNA level analysis, which focused on candidate genes picked in the DNA analysis, the significance threshold was set at 0.05. Empirical Bayes methods were used in assessing genome-wide significance (FDR) in statistical analysis of differential expression.

### Study approval

Patients/caretakers were consented to the study either before the sample collection (ethical permit code #R13109, and 2014/482) or retrospectively using samples stored in local hospitals or Uppsala Leukemia Biobank (#R19020). A written informed consent was received by the patient and/or caretakers. The study was approved by the regional ethical committees (Pirkanmaa Hospital District Ethical Committee, Finland, and Regional Ethical Review Board in Uppsala, Sweden). The study was conducted according to the principles of the Declaration of Helsinki.

## Supplementary information

Supplementary file

Supplementary tables

## Data Availability

The datasets generated and analyzed in the current study are available in Gene Expression Omnibus under the accession numbers GSE228632 (RNA-seq data), GSE49031 (array-based DNA methylation data, GSE227832 (RNA-seq data), GSE230295 (sc-RNAseq: CITE-seq+V(D)J-seq), and European Genome-phenome Archive under the accession number EGAD00001010164 (RNA-seq data), EGAD00001010128 (WGS data), EGAD00001011055 (sc genomics data). Single cell data from healthy BM B cells: https://bioinformatics.uef.fi/cells/public/HCA_Blineage/?ds=HCA_Blineage. CNVkit outputs from panel sequencing data are available in Zenodo under DOIs: 10.5281/zenodo.15167703, 10.5281/zenodo.15173882 and 10.5281/zenodo.15174016.

## Code availability

Code to reproduce key parts of the analysis can be accessed from GitHub: https://github.com/systemsgenomics/ETV6-RUNX1_Genomics_treatment_response.
