# Supplementary material for: Genomic determinants of therapy response in ETV6::RUNX1 leukemia
Source: Leukemia. 2025 Jul 9;39(9):2125–39. doi: 10.1038/s41375-025-02683-7 (PMC12380598; doi:10.1038/s41375-025-02683-7)
Supplement: Supplementary file 1 — Supplementary file [file 41375_2025_2683_MOESM1_ESM.pdf]

# Genomic determinants of therapy response in *ETV6::RUNX1* leukemia

Laura Oksa[1,2†], Sanni Moisio[3†], Khurram Maqbool[4], Roger Kramer[3], Atte Nikkilä[1], Buddika Jayasingha[3], Artturi Mäkinen[1,5], Hassan Foroughi-Asl[4,6], Samuli Rounioja[7], Janne Suhonen[3], Olga Krali[8,9], Miikka Voutilainen[10], Mari Lahnalampi[3], Kaisa Vepsäläinen[11], Sui Huang[3,12], Jesus Duque-Afonso[13], Julia Hauer[14,15], Jessica Nordlund[8,9], Valtteri Wirta[4,6], Olli Lohi[1,2*], Merja Heinäniemi[3*]

1. Tampere Center for Child, Adolescent, and Maternal Health Research, Faculty of Medicine and Health Technology, Tampere University, Tampere, Finland
2. Tampere University Hospital, Tays Cancer Centre, Tampere, Finland
3. The Institute of Biomedicine, University of Eastern Finland, Kuopio, Finland
4. SciLifeLab, Department of Microbiology, Tumor and Cell biology, Karolinska Institutet, Stockholm, Sweden
5. Department of Pathology, Fimlab Laboratories, Tampere University Hospital, Tampere, Finland
6. Genomic Medicine Center Karolinska, Karolinska University Hospital, Stockholm, Sweden
7. Department of Hematology, Fimlab Laboratories, Tampere University Hospital, Tampere, Finland
8. Department of Medical Sciences, Uppsala University, Uppsala, Sweden
9. SciLifeLab, Uppsala University, Uppsala, Sweden
10. Faculty of Biological and Environmental Sciences, University of Helsinki, Helsinki, Finland
11. Department of Pediatrics, Kuopio University Hospital, Kuopio, Finland
12. Institute for Systems Biology, Seattle, WA, USA
13. Department of Hematology/Oncology/Stem Cell Transplantation, Faculty of Medicine, University of Freiburg Medical Center, Freiburg, Germany
14. Department of Pediatrics, School of Medicine, Technical University of Munich, Munich, Germany
15. German Center for Child and Adolescent Health (DZKJ) and German Cancer Consortium (DKTK), partner site Munich, Germany

[†] Contributed equally, * co-senior authors

Corresponding authors: Merja Heinänimi, merja.heinaniemi@uef.fi, Olli Lohi, olli.lohi@tuni.fi, Laura Oksa, laura.oksa@tuni.fi

## Supplementary Material

### Supplementary Figures

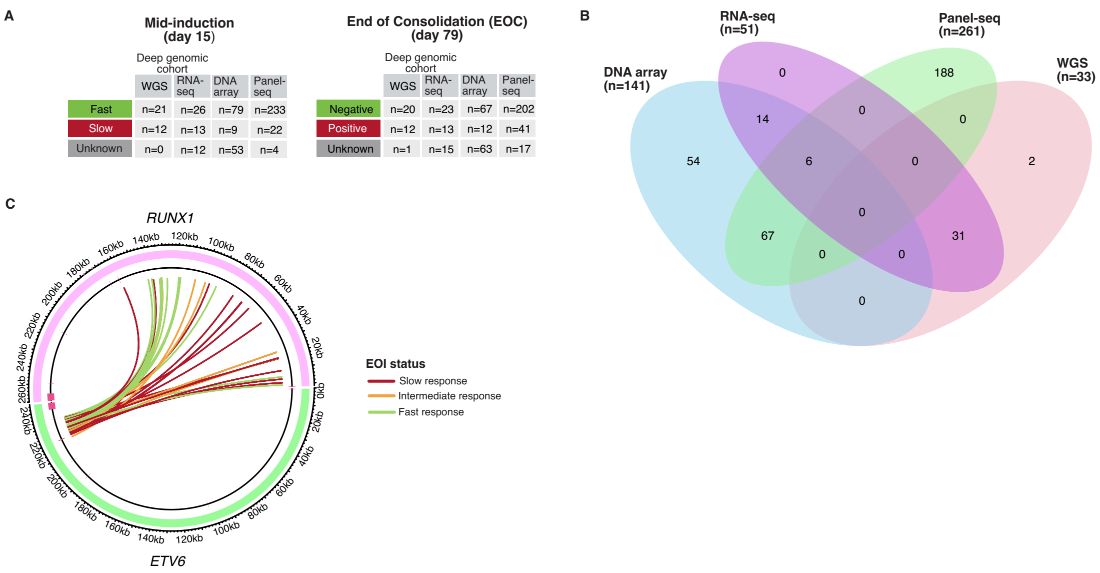


**Figure S1. Somatic DNA alteration patterns and used data types. A.** Number of cases profiled in each genomics assay by mid-induction and EOC responses. **B.** Circos plot showing *ETV6::RUNX1* fusion breakends by EOI response group (n = 33). Light purple color indicates *RUNX1*, and light green *ETV6* gene, respectively. The inner black line indicates introns, and the pink bars indicate exons. **C.** Venn diagram showing the overlap of data types used in the study.

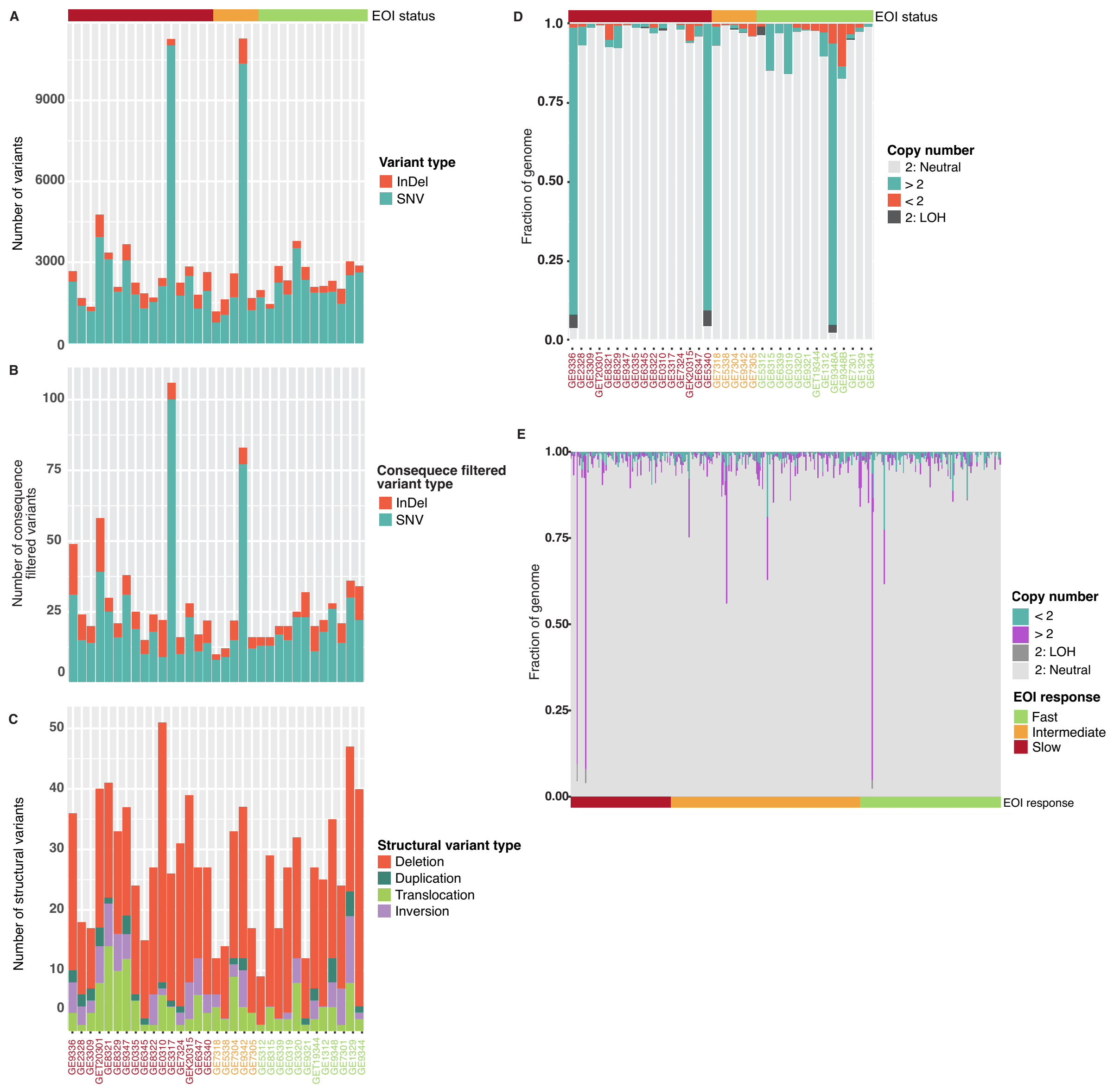


**Figure S2. Frequency of somatic alterations.** Bar plots representing the distribution of (**A**) all SNVs and InDels, (**B**) consequence-filtered coding and splice variant SNVs and InDels, (**C**) SVs, and (**D**) CNV fractions across the WGS samples (n = 33). The cases are organized by their MRD at the EOI. In the CNV WGS plot, codes GE9348A and GE9348B represent two separate leukemic clones of the same patient. **E.** CNV fractions across the whole CNV cohort (n = 358).

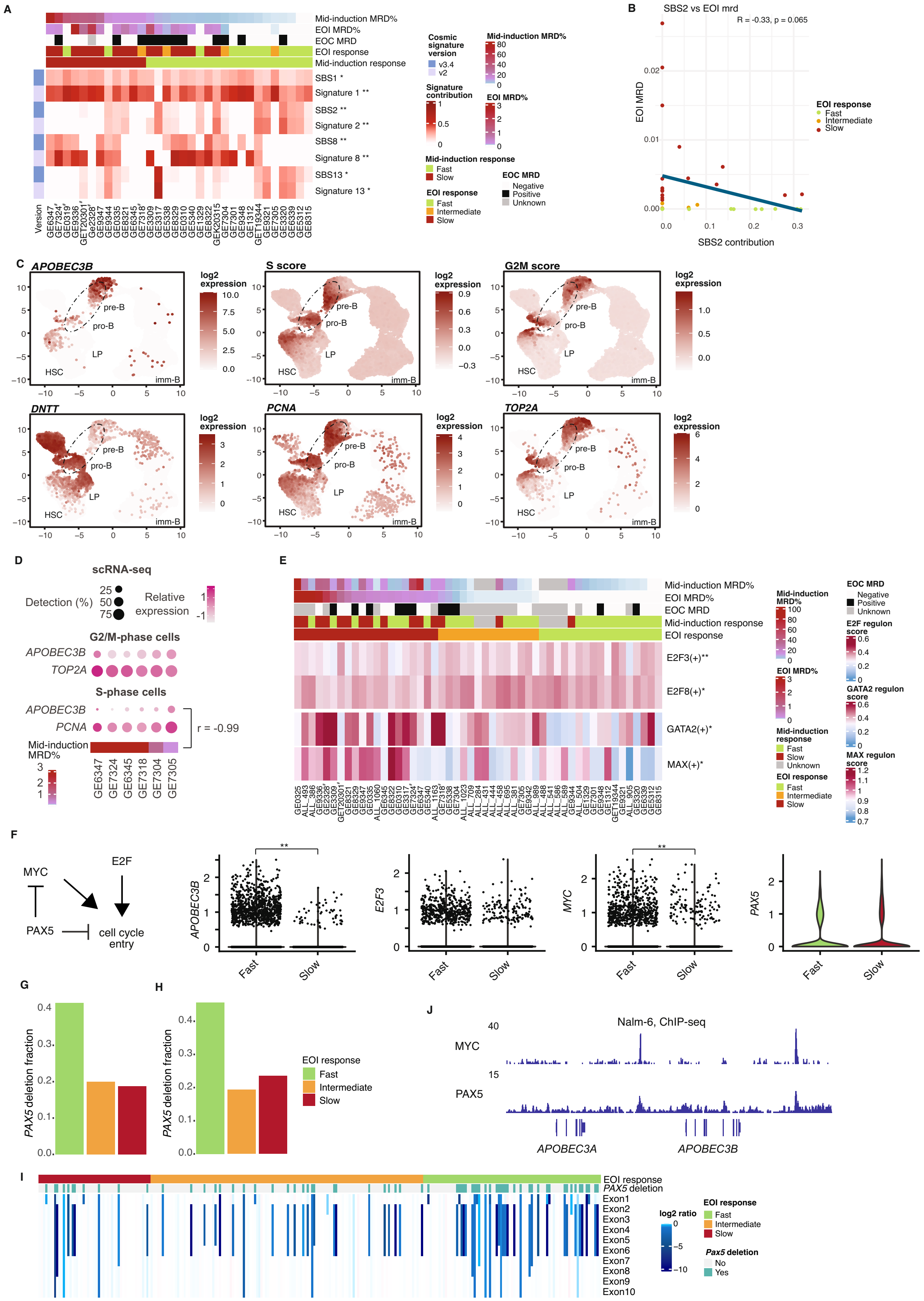

A
Mid-induction MRD%
EOI MRD%
EOC MRD
EOI response
Mid-induction response
SBS1 *
Signature 1 **
SBS2 **
Signature 2 **
SBS8 **
Signature 8 **
SBS13 *
Signature 13 *
Version
Cosmic signature version
v3.4
v2
Signature contribution
Mid-induction response
Fast
Slow
EOI response
Fast
Intermediate
Slow
EOC MRD
Negative
Positive
Unknown
B
SBS2 vs EOI mrd
R = -0.33, p = 0.065
EOI MRD
SBS2 contribution
EOI response
Fast
Intermediate
Slow
C
APOBEC3B
S score
G2M score
DNTT
PCNA
TOP2A
log2 expression
pre-B
pro-B
LP
HSC
imm-B
D
scRNA-seq
Detection (%)
Relative expression
G2/M-phase cells
APOBEC3B
TOP2A
S-phase cells
APOBEC3B
PCNA
r = -0.99
Mid-induction MRD%
GE6347
GE7324
GE6345
GE7318
GE7304
GE7305
E
Mid-induction MRD%
EOI MRD%
EOC MRD
Mid-induction response
EOI response
E2F3(+)**
E2F8(+)*
GATA2(+)*
MAX(+)*
E2F regulon score
GATA2 regulon score
MAX regulon score
F
MYC
E2F
PAX5
cell cycle entry
APOBEC3B
E2F3
MYC
PAX5
Fast
Slow
G
PAX5 deletion fraction
H
PAX5 deletion fraction
EOI response
Fast
Intermediate
Slow
J
Nalm-6, ChIP-seq
MYC
PAX5
APOBEC3A
APOBEC3B
I
EOI response
PAX5 deletion
Exon1
Exon2
Exon3
Exon4
Exon5
Exon6
Exon7
Exon8
Exon9
Exon10
log2 ratio
EOI response
Fast
Intermediate
Slow
Pax5 deletion
No
Yes

**Figure S3. Cell cycle, mutation signature, and regulon scores in different treatment response groups. A.** Mutation signatures analyzed with Musica (COSMIC v2) in light purple and with SigProfilerAssigment (COSMIC v3.4) in light blue stratified by the mid-induction MRD (n = 32). Two asterisks indicate a p-value < 0.05, and a single asterisk indicates a p-value < 0.1 (Spearman's rank correlation coefficient). **B.** SBS2 APOBEC mutation signature contribution and its correlation with EOI MRD presented as a scatter plot, with Spearman's correlation coefficient (n = 32). **C.** Expression of *APOBEC3B,* gene set scores for the S and G2M cell cycle phases, along with the expression of the marker genes for early B cells (*DNTT),* S-phase (*PCNA*) and G2M-phase (*TOP2A*) examined in healthy BM B cells (HCA scRNA-seq). The dashed regions correspond to cycling pro- and pre-B cells. **D.** Relative expression of the *APOBEC3B*, the S phase-specific gene *PCNA*, and the G2M-phase-spesific gene *TOP2A* in scRNAseq-data of leukemic cells in S and G2M cell cycle phase, respectively shown as dot plot heatmap (n = 6). Samples are ordered according to the mid-induction MRD level. **E.** The activities of the E2F3, E2F8, GATA2, and MAX regulons were compared in different responder groups, and classified using EOI response (n = 51). Case IDs with hash symbol indicates relapsed patients in panels A and E. Two asterisks indicate a p-value < 0.01, and a single asterisk indicates a p-value < 0.05 (Spearman's rank correlation coefficient). Abbreviations: EOI, end of induction; EOC, end of consolidation; HSC, hematopoietic stem cell; LP, lymphoid progenitor; pro-B, progenitor B cell; pre-B, precursor B cell. **F.** Schematic presentation of putative mechanism for cell cycle entry regulated by PAX5 based on previous functional studies (1,2). Violin plots show the expression levels of *APOBEC3B*, *E2F3*, *MYC,* and *PAX5* in S-phase cells in scRNA-seq data. The significance in expression detection rates is indicated by two asterisks (**), corresponding to an adjusted p-value < 0.01 ($\chi^2$ test). **G-H.** Bar plots representing deletions overlapping the *PAX5* locus (**G**) in the WGS cohort (n = 33) and (**H**) in the panel-sequencing cohort (n = 256), indicating more prevalent deletions in the

fast responder group compared to slow and intermediate responders ($p < 0.001$ according to Fisher's exact test for the combined cohort, n = 289). **I**. Heatmap presenting log2 ratios of each *PAX5* exon according to CNVkit (n = 256). *PAX5* deletion status is shown above the heatmap; each case with a deletion (based on the log2 cutoff < -0.4) overlapping any PAX5 exon is annotated to have a deletion. **J.** Chip-seq data showing the co-localized binding of PAX5 (GSE115764) and MYC (GSE168864) in the *APOBEC3B* locus in Nalm-6 cells.

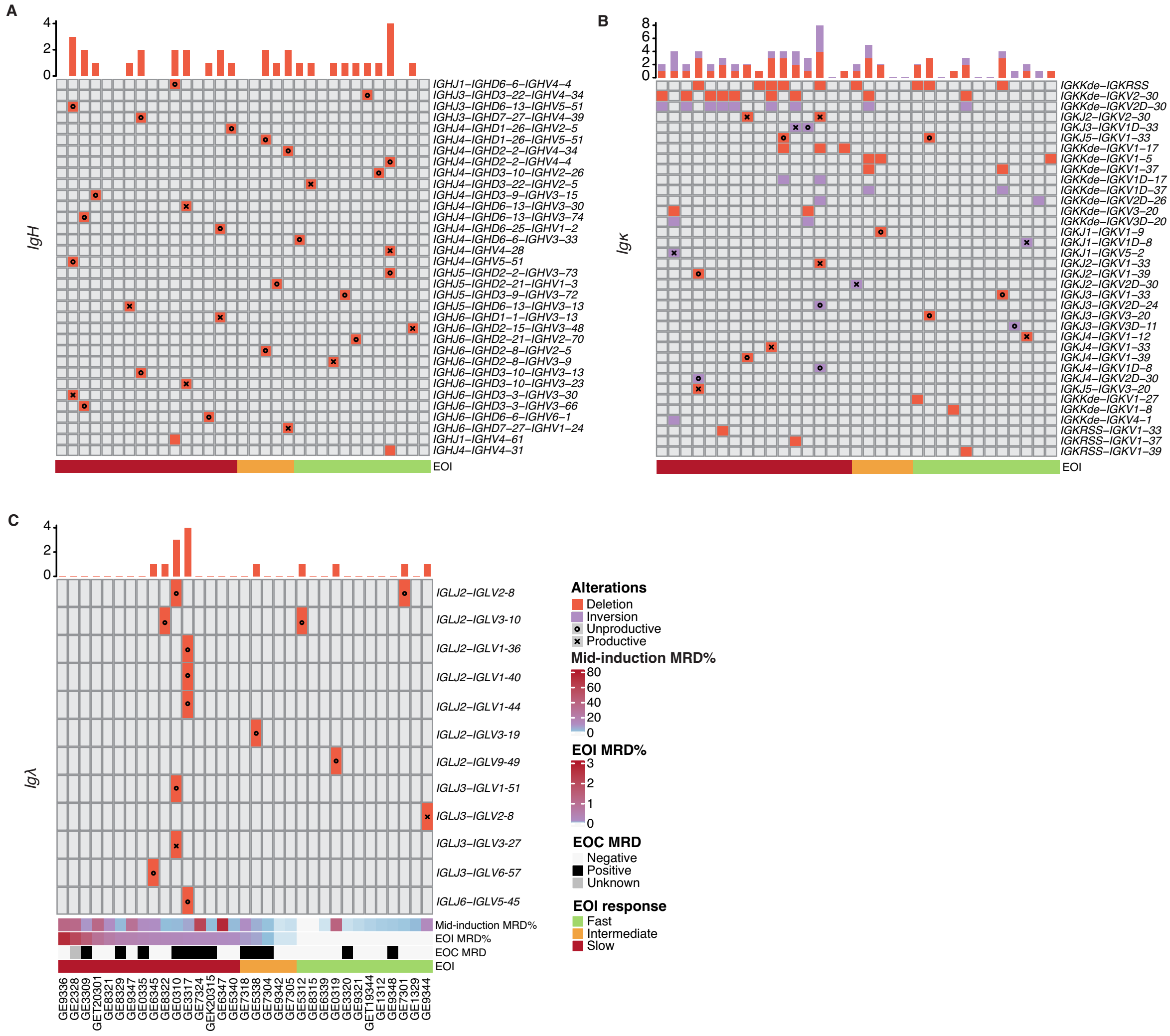


**Figure S4. Immunoglobulin gene rearrangements. A-C.** Oncoprints and bar plots showing the number of rearrangements affecting immunoglobulin heavy (*IgH*) and light chain (*Igk* and

*IgL*) genes in WGS samples (n = 33). The dot and dot with cross distinguish productive and unproductive alterations, respectively.

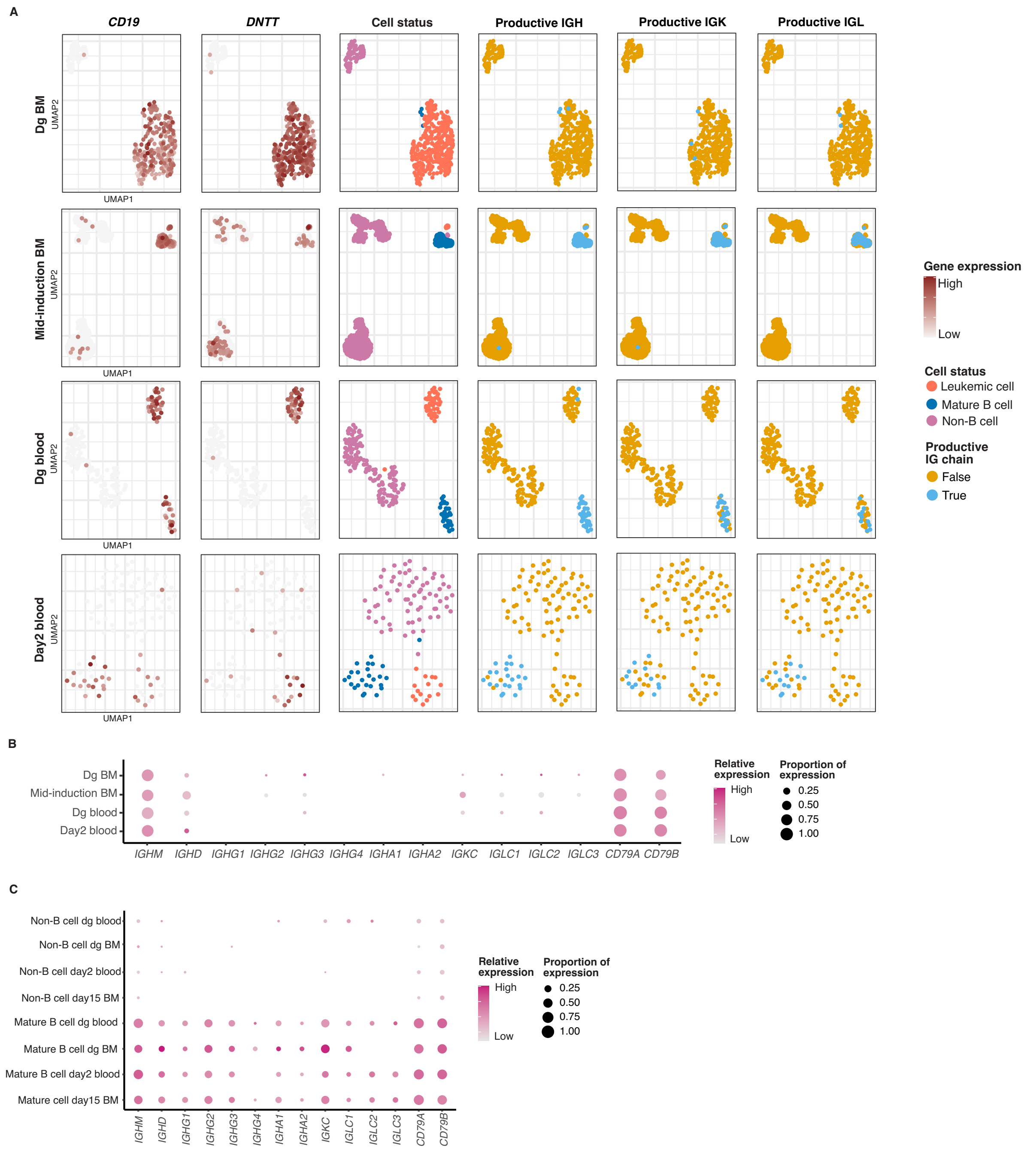


**Figure S5. Productive immunoglobulin chain rearrangements are enriched during induction therapy in a slow-responding leukemia. A-B.** Expression of B cell marker *CD19*, blast marker *DNTT,* and Ig heavy and light chains in scRNA-seq with integrated V(D)J sequencing data presented as UMAPs (**A**) and dot plot heatmaps (**B**) presenting expression

status at diagnosis, day 2 and at mid-induction (n = 1, GEK20315). The entire transcriptome was used to cluster cells in the UMAPs.

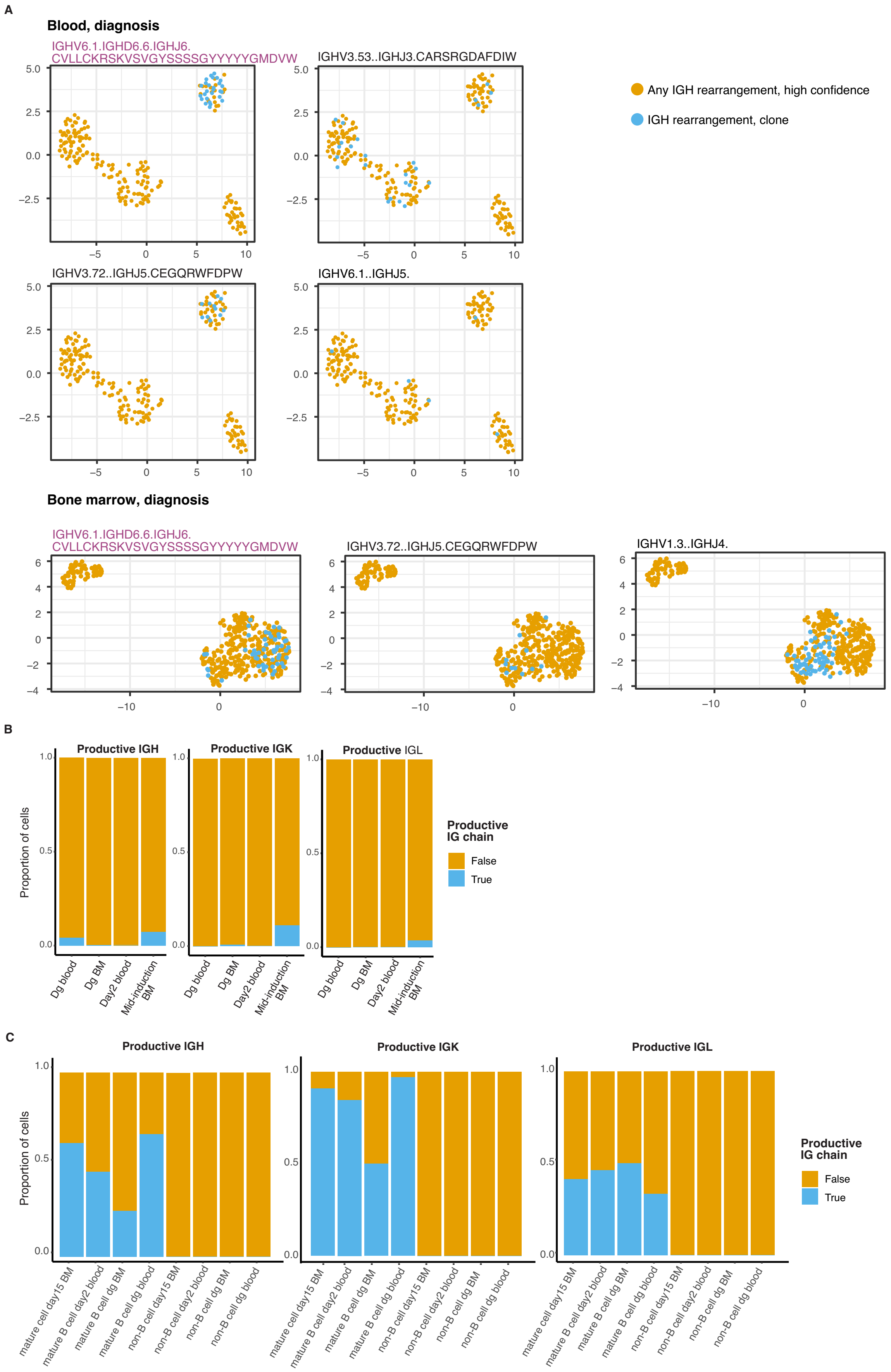
A
Blood, diagnosis
IGHV6.1.IGHD6.6.IGHJ6.
CVLLCKRSKVSVGYSSSSGYYYYYGMDVW
IGHV3.53..IGHJ3.CARSRGDAFDIW
IGHV3.72..IGHJ5.CEGQRWFDPW
IGHV6.1..IGHJ5.
Any IGH rearrangement, high confidence
IGH rearrangement, clone
Bone marrow, diagnosis
IGHV6.1.IGHD6.6.IGHJ6.
CVLLCKRSKVSVGYSSSSGYYYYYGMDVW
IGHV3.72..IGHJ5.CEGQRWFDPW
IGHV1.3..IGHJ4.
B
Productive IGH
Productive IGK
Productive IGL
Proportion of cells
Dg blood
Dg BM
Day2 blood
Mid-induction BM
Productive IG chain
False
True
C
Productive IGH
Productive IGK
Productive IGL
Proportion of cells
mature cell day15 BM
mature B cell day2 blood
mature B cell dg BM
mature B cell dg blood
non-B cell day15 BM
non-B cell day2 blood
non-B cell dg BM
non-B cell dg blood
Productive IG chain
False
True

**Figure S6. Clonal IGH rearrangements in leukemic cells and rearrangement status based on scVDJ-seq. A.** Clonal *IGH* rearrangements found from scVDJ-seq are shown on the UMAPs of diagnostic blood (upper panel) and bone marrow (lower panel) cells. Cells with high confidence *IGH* rearrangement are indicated in color (yellow: any, blue: cells with clone sequence). Purple indicates the same *IGH* rearrangement in both blood and BM samples. **B-C**. proportion of the cells with productive chain rearrangement status in leukemic (**B**) and non-leukemic cells (**C**). Mature B cells represent positive control for capture efficiency and non-B-lineage cells as negative control for ambient RNA background cells. Data in A-C encompasses scVDJ-seq data from one case (GEK20315).

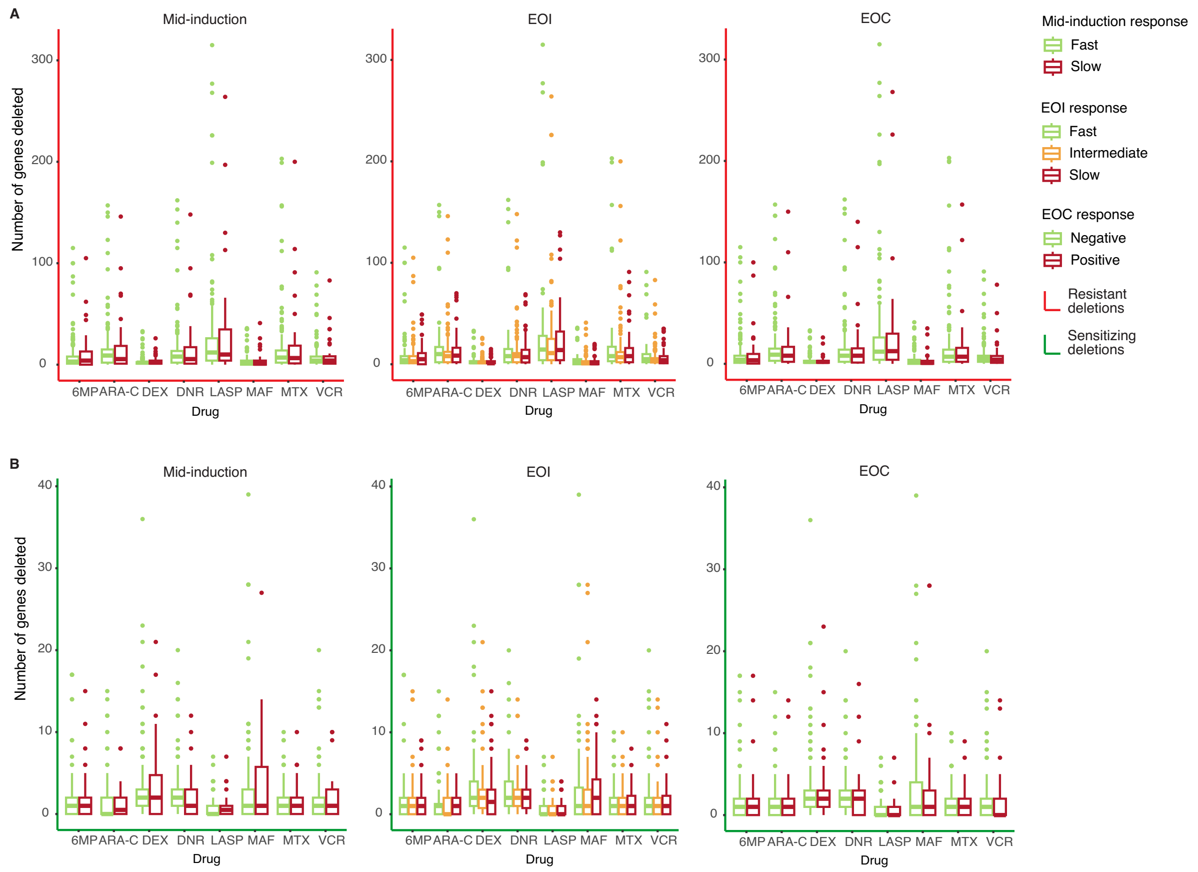


**Figure S7. Numbers of CNV deletions affecting drug response -associated genes from CRISPR screens. A-B.** Boxplots presenting the number of deletions affecting the genes associated with in vitro drug (**A**) resistance (red axels) or (**B**) sensitivity (green axels) by responder groups in all studied treatment time points: mid-induction, EOI, and EOC (total n = 358). The median number is indicated by the horizontal line, while upper and lower hinges represent the 75th and 25th percentile. Abbreviations: EOI, end of induction; EOC, end of consolidation; 6-MP, 6-mercaptopurine; ARA-C, cytarabine; DNR, daunorubicin; L-ASP, L-Asparaginase; MAF, maphosphamide; MTX, methotrexate; VCR, vincristine.

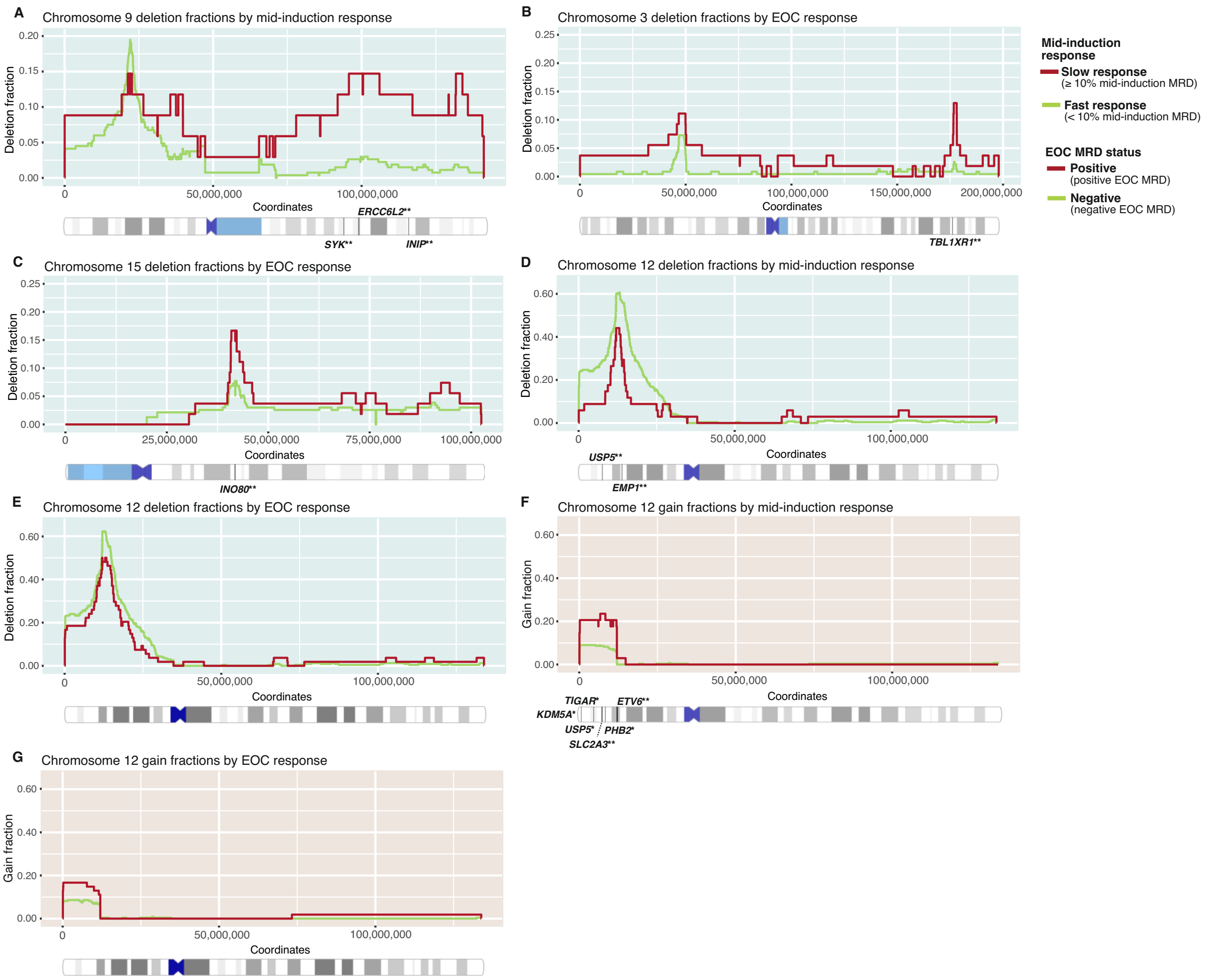


**Figure S8. CNV alterations in relation to treatment response. A.** Fraction of cases in mid-induction response group with a deletion of chromosome 9. In the EOC response plots, fraction of cases with (**B**) deletions of chromosome 3, and (**C**) deletion of chromosome 15. The fraction of cases in each response group classified using (**D**) mid-induction, and (**E**) EOC MRD with a partial deletion of chromosome 12. The fraction of cases in each response group classified using (**F**) mid-induction, and (**G**) EOC MRD with a partial gain of chromosome 12. In the mid-induction plots, red curves denote the fraction of slow cases with MRD ≥ 10 % (n = 34), and green curves indicate fast cases with MRD < 10 % (n = 267), harboring the indicated copy number change at each region of the chromosome. In the EOC response plots, red curves represent the fraction of MRD positive cases (n = 54), and green curves represent MRD negative cases (n = 233), harboring the indicated copy number change at each region of the

chromosome. The chromosome structure with relevant genes and their empirical p-values are visualized below the curves. Grey bars indicate cytobands and black bars indicate the location of highlighted genes.

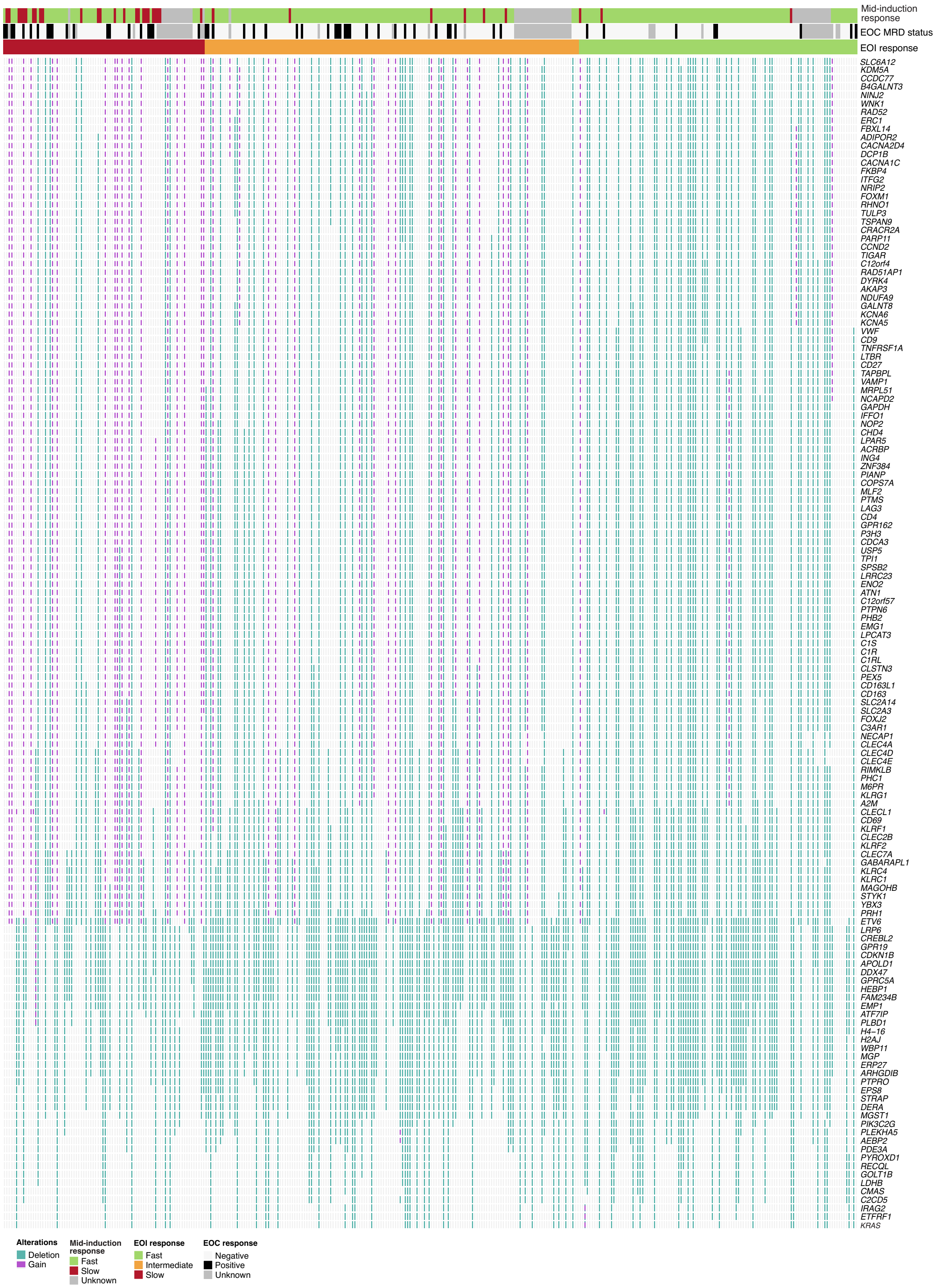


**Figure S9. Oncoprint visualizing EOI response group -differentiating CNV gains and deletions in chromosome 12 genes.** Genes are ordered according to chromosomal location

(gene nearest to chromosome 12 start at the top), and patients according to EOI response (n = 356). The EOI, mid-induction, and EOC response groups are annotated above the heatmap.

A

| Cell line | Mutation | Amino acid change | Position of STOP in WT/mut | Position (AA) of STOP in WT/mut | Prediction (Mutation taster) |
|---|---|---|---|---|---|
| REH KRAS KO14. | chr12:25380276_25380277delTG<br>chr12:25380271_25380272insAA | Q61Rfs*25<br>E63Lfs*6 | 567/255<br>567/204 | 189/85<br>189/68 | Disease causing<br>Disease causing |
| REH NT5DC1 KO10. | chr6:116432088_116432089insGT<br>chr6:116432089_116432104delTTGTCGGACACTGGAA | L112Vfs*36<br>L112Wfs*30 | 1368/441<br>1368/423 | 456/147<br>456/141 | Disease causing<br>Disease causing |
| REH NT5DC1 KO23. | chr6:116432089_116432090insGGCCG<br>chr6:116432089_116432090insGGGGC | L112Wfs*37<br>L112Wfs*37 | 1368/444<br>1368/444 | 456/148<br>456/148 | Disease causing<br>Disease causing |

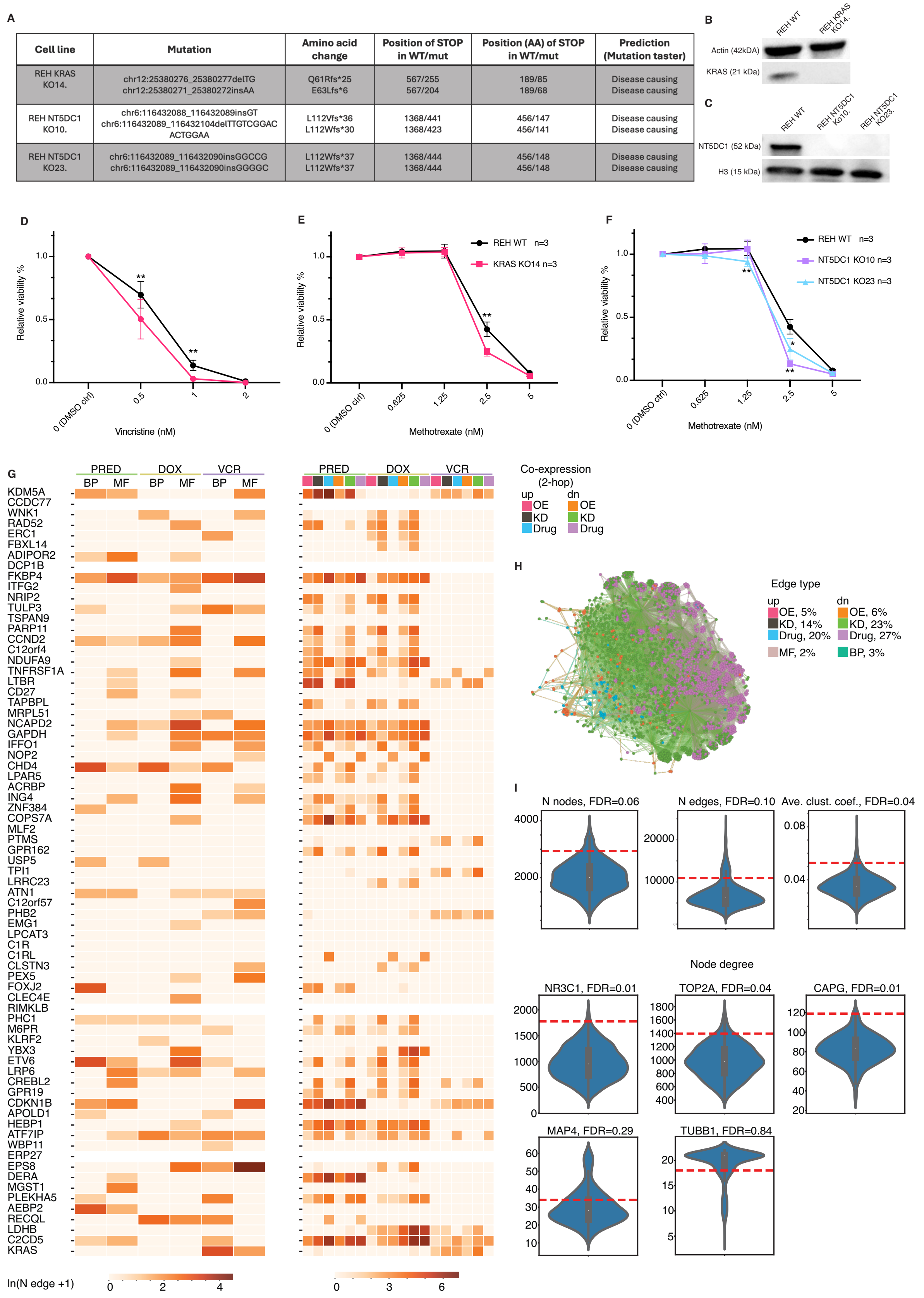


**Figure S10. Association of *KRAS* deletion and chromosome 12 CNVs to drug response A.**

REH-derived *KRAS* ja *NT5DC1* knockout cell lines were generated using CRISPR-Cas9. The

knockouts of KRAS and NT5DC1 proteins were confirmed with western blot (**B-C**). Quantifications compared to REH WT protein expressions were made: REH WT vs. KRAS KO14. = 0.04, REH WT vs. NT5DC1 KO10. = 0.00, REH WT vs. NT5DC1 KO23. = 0.02. Vincristine and methotrexate sensitivity of *KRAS* KO14. cells (**D-E**), and methotrexate sensitivity of *NT5DC1* KO cell lines (**F**) compared to REH WT cells. Drug treatments were made using three biological replicates per cell line. Two asterisks indicate a p-value < 0.01, and a single asterisk indicates a p-value < 0.05 (*t*-test). Abbreviations: EOI, end of induction; EOC, end of consolidation, DEXA, dexamethasone; DOXO, doxorubicin; PRED, prednisolone; VCR, vincristine. **G.** Heatmaps summarizing the number of paths from the drug target gene to the respective genes from chr12 (in rows) via one intermediate node. On the left, the intermediate nodes correspond to shared biological process (BP) or molecular function (MF) terms. On the right, the intermediate nodes are genes or compounds identified from perturbations assays that significantly regulate both the drug target and chr12 gene. The color tone corresponds to number of connecting edges in logarithmic scale. OE: overexpression, KD: knockdown. **H**. Network formed by the paths from G. **I.** Comparison of the network in H to networks obtained with randomly selected genes is shown as violin plots. The dashed red line corresponds to the metric score for the chr12 gene network. The empirical p-value for equal or high metric score based on the random network score distribution is indicated.

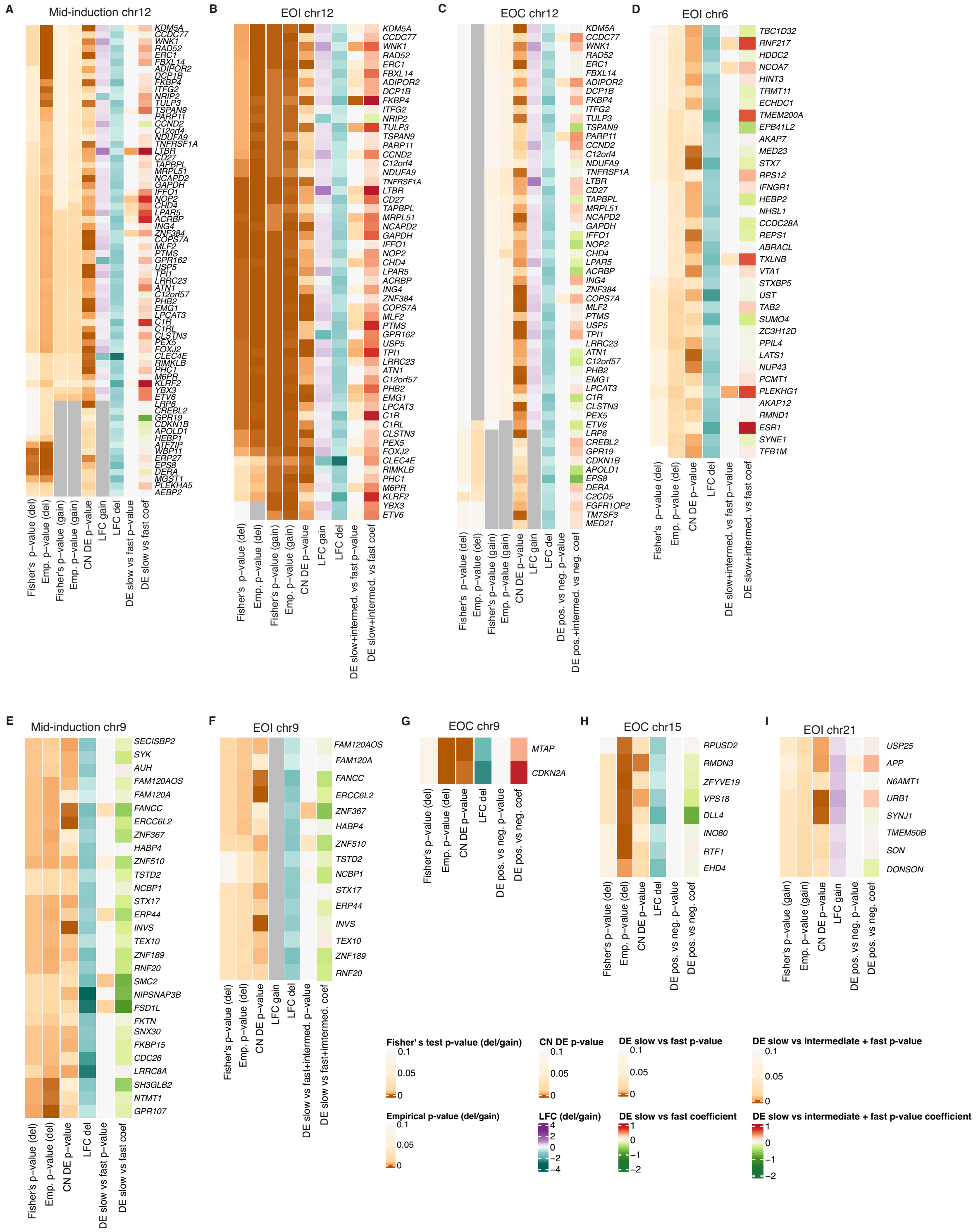


**Figure S11. Summary of genes affected by response-group differentiating CNVs, and their status in gene expression analyses.** The reported genes are expressed in leukemic cells (scRNA-seq) and arise from the indicated timepoint based on the Fisher's exact tests comparing deletion/gain prevalence between the respective responder groups and the respective copy

number status affects gene expression (DE analysis p < 0.05). Heatmaps are divided by timepoint and chromosome, visualizing genes arising from the respective response group comparisons: chromosome 12 and (**A**) mid-induction, (**B**) EOI (slow and intermediate vs fast), and (**C**) EOC, (**D**) chromosome 6 and EOI (slow and intermediate vs fast), chromosome 9 and (**E**) mid-induction, (**F**) EOI (slow vs intermediate and fast), and (**G**) EOC, (**H**) chromosome 15 and EOC, and (**I**) chromosome 21 and EOI (slow and intermediate vs fast) comparisons. Genes are ordered according to their chromosomal location (genes closest to chromosome start at the top), and for each gene, the heatmaps visualize the Fisher's exact test p-value, empirical p-value, p-value, and LFC from the DE analysis comparing the patients with different copy number status, and p-value and coefficient from the DE analysis comparing the respective responder groups. Abbreviations: DE, differential expression; del, deletion; Emp p-value, empirical p-value, EOI, end of induction; EOC, end of consolidation; LFC, log fold change.

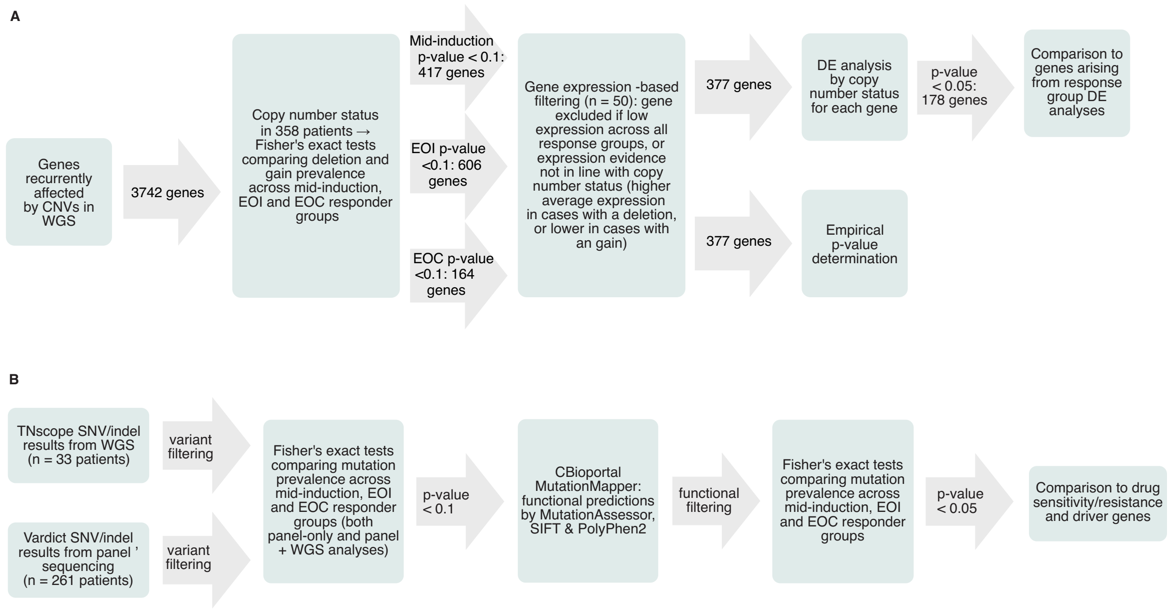


**Figure S12. Workflows for determining response-group differentiating genetic alterations.** Schematic workflow description for CNVs (**A**), and SNVs/InDels (**B**). Details are described in Supplementary Materials and Methods.

## Supplementary Materials and Methods

### DNA and RNA extraction

DNA samples were extracted from bone marrow or blood samples collected into PAXgene blood tubes (Qiagen GmbH, Hilden, Germany), or from primary ALL cells after Ficoll/Lymphoprep gradient separation using reagents in the QIAamp DNA Blood Midi kit (Qiagen), the AllPrep DNA/RNA/miRNA Universal Kit (Qiagen), the AllPrep DNA/RNA Kit (Qiagen), the Puregene Blood Kit (Qiagen), or GeneJET Genomic DNA Purification Kit Thermo Scientific). Remission samples were extracted using same reagents, but the sample was extracted from the blood or BM cells after remission.

RNA samples were extracted from bone marrow or blood samples collected into PAXgene blood tubes (Qiagen), or from primary ALL cells after Ficoll/Lymphoprep gradient separation using reagentes in the PAXgene Blood RNA kit, the AllPrep DNA/RNA/miRNA Universal Kit (Qiagen), the AllPrep DNA/RNA Kit (Qiagen), or the PureLink RNA Mini Kit (Thermo Scientific).

### WGS library preparation and sequencing

#### Batch 1: WGS library preparation and sequencing

Library preparation was done using 1.0 µg of genomic DNA as input and using the TruSeq DNA PCR-free library preparation kit (FC-121-3001/02, Illumina Inc.) according to the manufacturer's preparation guide #15036187. The genomic DNA was fragmented using a Covaris E220 system, aiming at 350-bp fragments. The ends of the DNA fragments were end-repaired and the 3' end adenylated to generate an overhang. Adapter sequences were ligated to the fragments via the A-overhang and the generated sequencing library was purified using AMPure XP beads (Beckman Coulter). The quality of the library was evaluated using the

FragmentAnalyzer system and a DNF-910 kit. The adapter-ligated fragments were quantified by qPCR using the library quantification kit for Illumina (KAPA Biosystems/Roche) on a CFX384 Touch Real-Time PCR instrument (BioRad) prior to cluster generation and sequencing. Sequencing was performed with paired-end 150-bp read length using v1 chemistry on a HiSeq X sequencing system (Illumina Inc.). Base calling was done on the instrument by RTA 2.7.1 and the resulting .bcl files were demultiplexed and converted to fastq format with tools provided by Illumina Inc., allowing for one mismatch in the index sequence.

**Batches 2, 3 and 4: WGS library preparation and sequencing**

WGS library preparation and DNA sequencing was performed by Novogene (HK) Company Limited (Hong Kong, China). Library preparation was done using NEBNext® Ultra™ DNA Library Prep Kit (New England Biolabs, MA, USA) following the manufacturer's recommendations and indices were added to each sample. The genomic DNA was randomly fragmented to a size of 350 bp by shearing, then DNA fragments were end polished, A-tailed, and ligated with the NEBNext adapter for Illumina sequencing, and further PCR enriched by P5 and indexed P7 oligos. The PCR products were purified (AMPure XP system) and the resulting libraries were analyzed for size distribution by Agilent 2100 Bioanalyzer and quantified using real-time PCR. DNA sequencing was performed in paired-end 150 bp mode using the Hiseq X or NovaSeq 6000 sequencing systems (Illumina Inc.). Base calling and the data demultiplexing were made using CASAVA (Illumina Inc.) base recognition and bcl2fastq softwares.

**Batch 5: WGS library preparation and sequencing**

Libraries were generated with NexSeq AmpFREE Low DNA Fragment Library kit (Lucigen Corporation) according to the instructions from the manufacturer. Briefly, 200 ng was

fragmented with Covaris E220 to an insert size of approximately 350 bp. The fragments were end-repaired, A-tailed and dual index adaptors (IDT for Illumina Unique Dual Indexes, product number 20022370 (Illumina) were ligated to the fragments, which were also subjected to double sided purification. Library quantification was performed by KAPA Library Quantification Kit (Roche). Sequencing was done on NovaSeq 6000 (Illumina) using paired-end 150 bp readout, aiming at > 90x coverage for tumor and > 30x coverage for normal samples. Demultiplexing was done using bcl2fastq2 Conversion Software v2.20 (Illumina).

**Targeted panel library preparation and sequencing**

Targeted panel sequencing was performed by using a custom panel design (Twist Bioscience, San Francisco, CA, USA), which contained DNA probes targeting the exons of selected genes (n = 874), recurrent SV breakend regions, as well as 3000 backbone probes (1 probe / 1 Mb) to facilitate genome-wide CNV discovery (see Supplementary Table 25 for all genomic regions covered by probes). Kapa HyperPlus (Roche, Basel, Switzerland) library preparation was utilized for targeted panel sequencing libraries of tumor samples (n = 261), and the remission samples for the panel-of-normals (PON, n = 38) using up to 250 ng input DNA. Gen Duplex Seq Adapter's containing unique molecular identifiers (UMIs) (Integrated DNA Technologies) were ligated to the fragments and indexing performed with dual-index (UDI) sample indexing primers (Integrated DNA Technologies). Purified, indexed libraries were equally combined before targeted capture with the custom DNA probes. Sequencing was conducted in paired-end 150bp mode using the NovaSeq X Plus sequencing method (Illumina Inc.), sequencing 80 M read pairs for tumor samples and 20 M read pairs for PON samples.

**RNA-sequencing analysis**

RNA sequencing libraries were prepared from 32 samples (31 matching to WGS samples) using the NEBNext Ultra Directional RNA Library Kit (New England BioLabs Inc.) with Globin-Zero gold (human/rat/mouse) or Ribo-Zero (human/rat/mouse) rRNA Removal Kits (Illumina Inc.). In addition, four samples were prepared with the Illumina TruSeq RNA Access library preparation kit (Illumina Inc.), six samples with Illumina TruSeq stranded Total RNA (RiboZero human/mouse/rat) kit (Illumina Inc.), and nine samples with the Script-Seq kit (EpiCentre). The libraries were paired-end (PE) sequenced (150bp) on an Illumina Hiseq 2500 or Novaseq 6000 instruments.

The raw sequencing data from 51 libraries in total were processed by utilizing the nf-core/rnaseq workflow v3.0 (3) based on nextflow v21.02.0.edge (4), which pipeline incorporates trimming of the paired-end reads by trimgalore v0.6.6, alignment to GRCh38.103 reference genome with STAR v2.1.6d (5). The Salmon tool v1.4.0 (6) was used to quantify the transcript abundance from the aligned reads, while these expression values were summarized to the gene level utilizing the Bioconductor tximeta v1.8.0 package (7). Subsequently, the data were analyzed in R by utilizing the EdgeR package v4.2.0 (8), normalizing the data by using the trimmed mean of M-values (TMM) method, also adjusting the data for library size, and finally performing log2 transformation for the counts per million (CPM) -scaled read counts, which values were used in the downstream analyses and visualizations of gene expression. Genes lowly expressed in all response group comparisons (mid-induction, EOI, EOC) were excluded by utilizing the FilterByExpr function, 15,279 genes remaining for further analysis. Moreover, differential expression (DE) analyses were conducted by utilizing the limma R package v3.60.2 (9), adjusting for batches (different library preparation methods) and determining the mean-variance relationship of the count data by using precision weights calculated by the voom function, while the lmFit function was used to fit a linear model to the

expression value of each gene. Finally, the eBayes function was utilized to perform the empirical Bayes moderation on the linear model fit. The different DE analyses conducted encompassed all the response group comparisons; mid-induction fast vs slow, EOI fast + intermediate vs slow and fast vs intermediate + slow, and EOC fast vs slow (n = 51); as well as copy number status comparisons for the 377 genes arising from the CNV analysis (n = 50, neutral vs deletion (if present) vs gain (if present)). Expression of the genes involved in S and G2/M phases during the cell cycle were studied as individual genes, but also gene set scores were calculated from normalized count matrix using GSVA ssGSEA v1.48.0 (10). Analyzed regulons were chosen from our earlier publication made by Mehtonen et al., (2020) (11). Gene, gene set, and regulon expressions were drawn using the ComplexHeatmap R-package (12). Correlation tests were made using Spearman's correlation by comparing gene set expression or regulon activity and MRDs (mid-induction and EOI). To calculate empirical p-values for S phase score patient's MRDs were randomized, and p-values calculated for 10, 000 times. Empirical p-value was calculated using formula: $(S+1)/(N+1)$, where S is number of p-values that were smaller than original p-value, and N number of the repeats.

**Alignment and variant calling**

Bioinformatic analysis of whole-genome sequencing data was conducted by using the BALSAMIC workflow v8.2.10 (https://balsamic.readthedocs.io/en/v8.2.10/) in the tumor-normal mode. The pipeline generated quality control metrics using FastQC v0.11.9 (13), trimmed adapter sequences and low-quality bases by using fastp v0.11.9 (14), and performed read alignment to GRCh37 reference genome by utilizing Sentieon v202010.02 (15), after which duplicated reads were marked by using Picard tools v2.25.0 (16). Moreover, the workflow visualized the quality control results by using MultiQC v1.11 (3) as well as performed variant calling by employing Sentieon (v202010.02) TNscope in identifying SNVs

and InDels, AscatNGS v4.5.0 (17) in identifying CNVs, and both Manta v1.6 (18) and Delly v0.8.7 (19) in identifying SVs.

Bioinformatic analysis of targeted DNA sequencing data was also done by using the BALSAMIC workflow v16.0.1 (https://balsamic.readthedocs.io/en/v16.0.1) similarly as described above except that the analysis was conducted in tumor-only mode (panel of normals, PON, included) and alignment performed by sentieon-tools (18). The workflow utilized the same bioinformatic tools mentioned above with the following version updates: fastp v0.23.2, Picard tools v2.27.1, MultiQC v1.22.3. In contrast to the WGS workflow, CNVkit v0.9.10 and VarDict v1.8.2 (20) tools were utilized in calling of CNVs, and SNVs/InDels, respectively. Regarding the CNVkit analysis, the targets in the bedfile were expanded to a minimum size of 100bp using a custom script. Next, these target and anti-target (genomic regions between the targets) files were generated from the the bedfile using the CNVkit python scripts. Coverage was calculated in the target and anti-target regions using cnvkit.py coverage using only reads with a minimum mapping quality of 20. Finally, the target and anti-target coverage files were combined and the PON used to correct the log2 ratio and to calculate the weight which is a metric of the reliability of the log2 ratio based on the spread of the log2 ratios of the normal samples using using cnvkit.py fix. The final reported regions correspond to combined longer CNV segments obtained using cnvkit.py segment --method cbs --drop-low-coverage.

**Variant annotation and filtering**

In addition to default TNscope filters, SNVs and InDels fulfilling the following technical requirements in the tumor sample were passed on to downstream analysis: sum of AD values (allelic depths for the ref and alt alleles) $\geq 10$ and $\geq$ AFDP (read depth to calculate allele fraction), alternative allele depth AD $\geq 3$, AF (allele fraction) $\geq 0.05$ and $< 1$, QUAL $\geq 40$ and/or PV2 (Fisher's exact test p-value for high-confidence reads) $\leq 0.05$, SOR (symmetric

Odds Ratio of 2x2 contingency table to detect strand bias) ≤ 3, and variant locating outside Encode Blacklist regions (23). Variant annotation was conducted by utilizing the nf-core/sarek v3.3.2 (based on Nextflow v23.10.0) workflow (24), that uses Ensembl VEP v110.0 (25) and reports one selected consequence per gene. These annotations were utilized to determine the higher impact coding variants, including all alterations with a high or moderate predicted impact in further analyses.

The SNVs and indels identified by VarDict in targeted panel sequencing data were filtered to encompass only mutations that pass the default tool filters, and which pass the cutoff of loqusDB curated frequency of observed variants (frequency < 0.01). To exclude technical artefacts, the VarDict results from the PON samples (n = 38) were utilized to exclude variants present in ≥ 5% of the normal samples (2 or more cases). These variants were further filtered based on allele fraction (AF, variants with AF ≥ 5% included) and strand bias (variant excluded if less than 1% of the evidence comes from the other strand or less than 5% of the evidence comes from the other strand and the total depth at variant position is less than 100 reads). The same VEP annotation and variant consequence filtering approach was used for the VarDict SNVs and InDels identified from targeted panel sequencing data as for the WGS mutations.

In addition to default Manta and Delly filters, the SVs identified by the tools in the WGS samples were further filtered to include only variants with at least 5 paired and split reads supporting the alternative allele in the tumor sample, also excluding variants overlapping with Encode Blacklist regions (23) by 50% or more, excluding deletions/insertions less than 150 bp in size (accounted as InDels), and excluding deletions and duplications more than 1Mb in size (accounted as CNVs). Collective SV lists were constructed by combining the filtered results from both callers using the SVDB v2.6.4 tool (26). In generating the union for each variant type; inversions, deletions, duplications, and insertions with 80% or more overlap and

translocation breakends within ≤ 5000 bp were regarded as the same event, prioritizing the breakpoints reported by Manta in such cases. AnnotSV v3.1.1 (27,28) was used to perform the annotation of the SVs, which were further annotated with consequences based on the following criteria: deletions overlapping 100% with the coding region of a gene were annotated with a loss consequence, duplications overlapping 100% with the coding region of a gene were annotated with a gain consequence, deletions and duplications overlapping 1–99% with the coding region of a gene and translocation and inversion breakends locating between the transcript start and end coordinates were annotated with a coding sequence variant consequence. Variants fulfilling any of these criteria constituted the list of coding SVs, while variants annotated with a gene hit but not passing these criteria were annotated as noncoding variants. For variants with no gene annotations, the closest genes both upstream and downstream were determined by bedtools v2.30.0 (22), and those genes were annotated with an intergenic variant consequence.

The results from AscatNGS were also filtered by size (events > 1 Mb in size accounted as CNVs) and by removing events that overlapped 50% or more with the Encode Blacklist regions (23). Accordingly, SVDB tool was used to combine the deletions and duplications with size greater than 1 Mb, identified by AscatNGS, Manta, and Delly to generate the collective CNV calls, using the same 80% overlap criterion to account for the event as the same one and prioritizing Manta breakpoints over Delly over AscatNGS. The merged CNV results were annotated using AnnotSV, and the variant consequences were further annotated by using the same criteria as for SV deletions and duplications. Events reported with a copy number of 2:0 (total copy number:minor copy number) by AscatNGS were classified as copy number neutral loss-of-heterozygosity events (LOH). To yield similar CNV calls from the targeted panel CNVkit results, the log2 values were used to determine gains (log2 of the segment ≥ 0.3) and deletions (log2 of the segment ≤ -0.4) reflecting cutoffs for high purity and typical ploidy 2

estimated based on cases with this clinical information available. The nearby regions were merged if they presented the same copy number change (gain/deletion) and were ≤ 10,000 bp apart. In addition, a similar filtering approach was utilized as for AscatNGS CNVs, i.e., only events > 1 Mb in size were regarded as CNVs, and events overlapping 50% or more with Encode Blacklist regions were excluded. Regarding the *PAX5* gene locus where the panel included probes at each exon and recurrent breakpoint, all deletions identified by CNVkit from the panel sequencing data (log2 of the segment ≤ -0.4) overlapping the locus were determined without the size filter to also include focal deletions in the analysis.

**Analysis of immunoglobulin gene rearrangements**

Immunoglobulin (IG) gene rearrangements were analyzed by utilizing the IgCaller tool (v1.2.1) (29) providing tumor and normal bam files as input and setting the tumorPurity parameter based on the consensus estimates of blast percentage. The filtered output file from the tool was further filtered to include only events with minimum of 5 split reads and/or abnormal insert size reads supporting the rearrangement. Functionalities estimated by IgCaller were primarily used in further analyses, but the functionalities were also annotated by using IMGT/V-QUEST (30) and IgBlast (31) for rearrangements with provided sequences.

**Copy number analysis using methylation array data**

Methylation data (32,33) was analyzed in R version 4.1.2 using Bioconductor (v3.14) packages methylumi (v2.40.1) (34) and conumee (v1.28.0) (35) including dependencies minfi (v1.40.0) and DNAcopy (v1.68.0). Methylumi reads the primary methylation data ("IDAT" files) and provides background correction and normalization using Illumina controls. Conumee calls copy number variations after fitting data to a set of normal reference samples for which 86 normal samples from GEO dataset GSE49031 were used. Script defaults were used for all

processing; no non-default parameters were required or provided, and the reference genome version was GRCh37.

Segments with median signal ≤ -0.05 were classified as deletions, segments with median signal ≥ 0.1 were classified as gains, and regions not fulfilling these criteria as copy number neutral. In RNAseq expression data that was available for a subset (n = 51) of patients, putatively deleted regions were also found to be enriched in mono- and nearly mono-allelic expression as would be expected, which supported our choice of thresholds. Additionally, events overlapping with Encode Blacklist regions (23) by 50% or more were excluded from further analysis. Events > 1 Mb in size were accounted as CNVs in downstream analyses.

**Determination of response-group differentiating gene alterations**

To distinguish mutations that might differentiate the responder groups, Fisher's tests were conducted to compare the prevalence of coding SNVs/indels that affect each gene included in the targeted panel (Figure S12B). The tests were conducted for each timepoint comparison (mid-induction, EOI and EOC responder groups), and for both panel-only (n = 261) and panel + WGS (n = 294, only genes affected in both cohorts included) mutations. The genes for which the p-value from any of these comparisons was below < 0.1 (excluding B cell receptor and T cell receptor genes, n = 80) and which were mutated in at least 3 patients were included in further analysis, and cBioPortal MutationMapper tool (36,37) was utilized to predict the functional consequences for these variants. The mutations were further filtered based on these predictions, including only nonsense and frameshift ones in addition to variants with high impact prediction by MutationAssessor (38) and/or any deleterious annotation (deleterious, deleterious_low_confidence) by SIFT (39) and/or probably/possibly damaging prediction according to PolyPhen2 (40) to yield a list of variants more likely to impact the function of the encoded protein (Supplementary Table 19). Similar Fisher's exact tests were conducted for

genes with functionally filtered variants in 3 or more patients, and genes with significant differences in functionally filtered variant prevalence ($p < 0.05$) are reported and compared to ALL driver genes (41) and genes arising from the CRISPR-based drug-gene interaction screens by Oshima et al., (2020) (42) and Poulard et al., (2019) (43).

To study response-associated CNVs, CNV data was combined from the WGS (n = 33), targeted panel (n = 255) and array-based (n = 70) analyses (total n = 358) (Figure S12A). 72 patient samples were included in both methylation array and targeted panel analyses, and for 70/72 of these patients, the panel CNV data was used, while for the 2 cases, the CNV calls from the targeted panel seemed unsuccessful, so the array CNV calls were used downstream.

Genes recurrently affected by CNVs (affected in five or more patients altogether and/or affected by deletions and/or gains in three or more patients, excluding genes on X and Y chromosomes, n = 3742) in the WGS cohort were included in further analysis, and Fisher's exact tests were computed to compare the prevalence of CNVs affecting these genes among the different responder groups. Both deletions and gains and the response categories at each timepoint (mid-induction, EOI and EOC) were assessed separately. In each analysis, if the gene was affected by the present alteration type in five or less patients with MRD information at that time point, it was excluded from further analysis. The EOI response groups comparisons were conducted both by grouping fast and intermediate groups and comparing against slow, as well as by grouping intermediate and slow groups (MRD positive) and comparing against MRD negative fast responders.

The top response group -differentiating gene hits were chosen for further analysis based on the p-value from Fisher's exact tests: $p < 0.1$ from either day 29 comparison, mid-induction or EOC response group comparisons. For a subset of cases (n = 50), RNA-seq data was available and was used to exclude genes with low expression across all response groups, and to determine average expression in cases with a deletion/gain vs without, including genes for which the

average expression in the cases affected by the present variant type was in line with the copy number status (lower average expression in cases with deletions vs others, higher average expression in cases with gains vs others), in further analyses (n = 377).

To assess whether the response group differences at the top-ranking CNV loci would be likely to arise randomly, empirical p-values were determined by randomizing both CNV coordinates and response groups specific CNV events and analysed loci included in the analysis are described in Supplementary Table 23. Coordinates of these CNVs of interest (3, 9, 12p, and 15 deletions, 10, 12, and 21q gains) were randomized 10,000 times utilizing bedtools shuffle, shuffling them to a random location on the same chromosome (or specified chromosomal region on the same chromosome described in Supplementary Table 23) maintaining the size of the event and not allowing possible multiple events in the same sample to overlap in the shuffled output. Furthermore, for each of the 10,000 iterations of shuffled CNV coordinates, the patient response groups were also randomized, maintaining the overall number of cases representing each responder group. From this randomized CNV data, the response group difference at the gene loci of interest (n = 377) were computed for all 10,000 iterations, and compared to the difference seen in the not-randomized real data, yielding the empirical p-value for each gene locus (fraction of the randomized iterations that result in a difference equal to or greater than the real observations). In addition, chromosome 16 gains were included in the analyses as controls.

In addition, the top resistance or sensitivity inducing genes according to the CRISPR screens by Oshima et al., (2020) (42) and Poulard et al., (2019) (43) were determined and compared to the identified CNV deletions. The significant genes ($q \leq 0.1$) affecting dexamethasone sensitivity or resistance according to Poulard et al., (2019) (43) were included in the comparison, while the top genes from the vincristine, daunorubicin, L-asparaginase, maphosphamide, methotrexate, cytarabine, and 6-mercaptopurine screens by Oshima et al.,

(2020) were determined by multiple cutoffs; top 100 and top 200 genes according to the gene rank, and the top genes according to FDR (< 0.25). To test if the response groups differ in the number of patients that harbor deletions of these top sensitivity/resistance associated genes, Fisher's exact test was utilized to compare the number of cases in each responder group that harbored any deletion affecting the top genes vs the number of cases without any such deletion. In addition, the median prevalence of deletions affecting the top sensitivity/resistance associated genes in the different responder groups was determined. To test if such differences would be likely to arise with any set of genes, empirical p-values were calculated by selecting the same number of random genes (1000 iterations) and determining the differences in the median numbers of deletions affecting these genes between the responder groups and comparing these values to the ones from the real gene set. These statistical analyses were conducted for each timepoint comparison (mid-induction, EOI, and EOC), each drug screen and each top gene cut-off (Supplementary Table 16).

The *PAX5* locus was assessed separately, as both focal (< 1 Mb in size) and CNV (> 1 Mb in size) deletions overlapping the gene region were considered from both WGS and panel sequencing cohort (total n = 289). To assess if these deletions are associated with the EOI response, Fisher's exact test was conducted comparing the fast responders to slow and intermediate responders.

**Single cell genomics from primary ALL cells**

To evaluate genomic features detected in bulk profiles at cellular resolution bone marrow or blood mononuclear cells collected at diagnosis, at day 2 and at mid-induction (day 15) were analyzed from cryopreserved samples. Libraries were prepared for transcriptome, cell surface protein marker (ADT) and BCR VDJ-rearrangement analysis. Cells were thawed in a 37°C water bath, immediately after adding 0.5 ml RPMI 1640 media (Thermo Fisher Scientific) +

10% FBS (Gibco) + 20 µl DNAse (Roche 100U/µl) on top, then moving cells to 15 ml Falcon and filling the volume drop-wise using gently swirling. For samples with < 1 million cells, 10 µl of DNAse was added, filling volume to 5 ml. Cells were washed two times, then centrifuged and re-suspended in 50 µl Cell Staining Buffer (BioLegend, San Diego, CA, USA) for counting. Subsequently, 100,000 – 200,000 cells per sample were processed by blocking with 5 µl of Human TruStain FcX Blocking Solution (BioLegend) for 10 min at 4°C. Cells were stained with surface marker antibody pool (0.25 µg per million cells per specific antibody, except 0.125 µg for CD45 and CD8) and using 0.1 µg of hashtag antibodies to allow multiplexing two samples per lane, incubating for 30 min at 4°C. Samples were washed three times with cell staining buffer, counted and assessed for viability using trypan blue staining, and sample pools prepared, centrifuged and loaded to Chromium lanes (10x Genomics). The following kits were used for library preparations: Chromium Next GEM Single Cell 5' Library & Gel Bead Kit v1.1, Chromium Single Cell 5' Library Construction Kit, Chromium Single Cell 5' Feature Barcode Library Kit Chromium Single Cell V(D)J Enrichment Kit, Human T Cell Chromium Single Cell V(D)J Enrichment Kit, Human B Cell. If post-stain viability was > 70% for all samples, equal ratios were loaded aiming at 10 000 cells. Otherwise, loading ratio was adjusted such that more cells were analyzed from the sample with better quality. The 5' gene expression, ADT- and VDJ (BCR) libraries were prepared following manufacturer instructions (10x Genomics) and cDNA qualities assessed using Bioanalyzer. Libraries were index barcoded and sequenced using Illumina Novaseq.

**Single cell genomics data processing**

Primary ALL scRNA- and ADT-seq data and reads from from VDJ (BCR) sequencing were aligned with Cell Ranger 6.0 version to human reference (GRCh38). The transcriptome data was first run with default settings, followed by expected number of cells set to 10 000

(parameter force-cell). Downstream analysis was performed in R using functions available in Seurat (v4), DSB, qqplot2 and dittoSeq packages. Cells with antibody aggregates were detected based on high ADT count (> 10 000) and discarded. Background droplet signal was used for normalization of hashtag and cell surface marker signals. First, droplets not assigned as cells and with at least one RNA and protein count were included from raw count matrices. For background signal fit, median and median absolute deviation (MAD) metrics were calculated to define candidate background and cell-containing (from Cellranger filtered output) droplets. In addition, droplets with > 25% mitochondrial reads were filtered out. From cell-containing droplets cells with low gene count and > 10% mitochondrial reads were discarded. Donor (and singlet/doublet) assignment was carried out based on two independent analyses: i) background-scaled (DSBNormalizeProtein, denoise.counts=F, use.isotope.control=F) hashtag signals and ii) clustering cells by single nucleotide-polymorphisms (SNPs) detected using cellSNP-lite (v. 1.2.3) using parameters minMAF = 0.1, minCount = 20 followed by vireo (v.0.5.8) analysis with seed=42 (44). Demultiplexing results were highly concordant between the tools. The BCR vdj metadata was retrieved from CellRanger outputs. scRNA-seq raw counts were normalized to 10 000 counts per cell and log-converted prior to downstream clustering and dimensionality reduction analyses. Strict filtering step was designed to ensure cells compared for the VDJ-repertoire represent good quality singlet data: i) doublets were detected and removed at sample pool level by scDblFinder_1.4.0 by providing snp-based known doublets as prior information to scDblFinder function. At sample level, i) 5% cells with lowest nGenes, or highest mitochondrial count percentage, or RNA library size < 1500 were flagged low quality; ii) cells assigned to 20% weakest quality by all three criteria were flagged low quality (lowest nGenes, RNA library size; highest mitochondrial count percentage). iii) Louvain algorithm was run on neighborhood graph calculated based on PCA coordinates (1:50) defined based on 3000 most variable genes and this initial clustering with number of neighbors 15, resolution 0.5 used to

perform cluster-wise outlier detection, flagging cells with the highest and lowest nCounts and nGenes, outliers defined by MAD cutoff 2. As a final step, the scDblFinder function was re-run at sample level. The QC flagged cells were discarded and higher resolution clustering (resolution = 2, nNeighbors = 3, nTrees = 10, cosine distance) and projecting data to UMAP (nNeighbors = 30) were used to separate leukemic (MME+, DNTT+, CD19+) from non-leukemic mature B and non-B-lineage cell populations based on examining marker genes (*IGLL1, VPREB1, DNTT, MME; CD34, HBA1, CD3D, CD4, CD8A, GZMB, GZMK, HLA-DQA, PTPRC*). The productive IGH, IGK and IGL events were retrieved from CellRanger annotations (https://10xgenomics.github.io/enclone/pages/auto/features.html) requiring that the event was classified high confidence (unlikely to be a chimeric sequence or other artifact) with min 5 supporting UMIs. Clonal recombination events were analyzed by retrieving the information about the respective V (D) and J-chains and their CDR3 sequence. Characterization of gene expression in normal bone marrow B-lymphoid cell states was performed using data from healthy donors, available from the HCA data portal. Detailed description of sample processing can be found from our earlier publication by Mehtonen et al., (2020) (11).

**Differential distribution of read counts: scDD analysis**

The gene expression distribution in S-phase cell cycle score between the fast (n = 2) and slow responders (n = 4) (mid-induction classification), were analyzed with the scDD package (45,46). The tool enables comparisons based on differential distribution and proportion of zeros between two groups of cells. For those genes that do not show DDs in the nonzero values (DE, DM, DP categories), scDD allows a user to evaluate whether the proportion of zeroes differs significantly between the two conditions (DZ category) based on a $\chi$ 2 test *p*-value of less than 0.025 (after adjustment for multiple comparisons using the method of (47))

**CRISPR-Cas9 mediated genome editing and *in vitro* drug treatments**

REH cells were cultured in RPMI media (Gibco), supplemented with 2 mM L-glutamine (Gibco), 1 % antibiotics (0.5 U/ml penicillin and 0.5 µg/ml streptomycin; Lonza, Basel, Switzerland), and 20 % FBS (Gibco) in standard cell culture conditions, 37°C in 5% CO2, and split every 3 days. REH derived *KRAS* and *NT5DC1* knockout cell pools were obtained from Synthego (Synthego Corporation, Menlo Park, CA, USA). The bulk cells were single-cell sorted into 96-well plates in conditioned media containing 40% of media harvested from unmodified and modified REH cells and 60% of RPMI-1640 media. All cell lines have been tested and proven regularly negative for mycoplasma, and STR genotyping was performed in Eurofins Genomics (Ebersberg, Germany) to authenticate the REH cell line.

Single-cell clones were screened for mutations with T7 endonuclease (New England Biolabs, Ipswich, MA, USA) and confirmed by Sanger sequencing (DNA sequencing and genomics service, University of Helsinki, Helsinki, Finland). MutationTaster2 (48) was used to study the functional effects of the mutations and changes in amino acid sequences and to predict the disease-causing potential. The silencing of KRAS and NT5DC1 at the protein level was confirmed using Western blot. Shortly, for protein extractions, cells were lysed with CelLytic M reagent (Sigma Aldrich, Saint Louis, MO, USA), according to the manufacturer's protocol. Protein samples were loaded on Mini-PROTEAN® TGX Stain-Free™ Precast 10% or 12% gels (BioRad, Hercules, CA, USA) and transferred to 0.2µM PVDF membrane using Trans-Blot Turbo Transfer Pack and Trans-Blot Turbo transfer System (BioRad). Membranes were processed using standard procedures, with KRAS (1:1000 in 3% BSA, #33917S, Cell Signaling Technology), NT5DC1 (1:2000 in 3% BSA, #CF501589, Origene) and Histone H3 (1:10,000 in 3% BSA, #9715S, Cell Signaling Technology) primary antibodies, and Horseradish peroxidase conjugated anti-rabbit (1:5000 in 3% BSA, #7074, Cell Signaling Technology) or anti-mouse (1:5000 in 3% BSA, #7076, Cell Signaling Technology) secondary antibodies.

Amersham ECL Reagent (GE Healthcare) was used for chemiluminescence reaction and chemiluminescence was detected with ChemiDocTM XRS+ using Image LabTM Software (BioRad). PageRuler Plus prestained protein ladder (Thermo Fisher Scientific) was used as a reference for protein size. ImageJ was used for quantification of protein expression (49).

To study the methotrexate sensitivity of the *KRAS* and *NT5DC1* KO cell lines, 10,000 REH wildtype and REH knockout cells were plated per 96-well plate. Cells were treated with methotrexate (0.625, 1.25, 2.5 and 5 nM; #13960 Cayman Chemicals), or vincristine dilution series (0.5, 1, 2 nM, #V8879, Sigma), and incubated for 96 hours at 37°C in 5% CO2. Noteworthily, in methotrexate assays, dialyzed FBS (#A3382001, Gibco) should be used to avoid the methotrexate protection by small molecules, such as thymidine and hypoxanthine (50–52). After incubation, cell viability was measured using CellTiter-Glo assay (Promega, Madisson, WI, USA).

***Ex vivo* drug screen**

Bone marrow samples were collected at diagnosis in heparinized glass tubes and analyzed in Uppsala University (Sweden). *Ex vivo* drug responses (dexamethasone, doxorubicin, vincristine, prednisolone) were assessed by the fluorometric microculture cytotoxicity assay (FMCA) (53), and the experimental procedures including handling of ALL samples followed Frost et al., (2003) (54,55). In brief, FMCA generates a survival index (SI%) and a high numerical SI% indicates *ex vivo* resistance to the drug.

For dexamethasone-only treatments, MNCs were thawed using an in-house method, and 20,000 cells of each patient were plated per well. Cells were treated with dexamethasone (0-25 nM; D8893; Sigma-Aldrich, St. Louis, Missouri, USA), in increasing concentration range of 0-25 nM. Cells were incubated at 37°C in 5% CO2, and cell viability was measured after 48 h

incubation using CellTiter-Glo assay (Promega, Madisson, WI, USA). The results were normalized using DMSO-treated cells to obtain relative viability.

**Analysis of protein and gene interactions**

We used SPOKE (Scalable Precision Medicine Open Knowledge Engine, v. 2021), a knowledge graph (56) and a multi-omics graph database that has ingested molecular, regulatory, causal and ontological relationships in biomedical entities to build a network of > 5 million network nodes, to retrieve direct drug target proteins, protein-protein interactions (directed edges), and to investigate the network of gene-level relationships among the 73 genes of interest from chromosome 12. We complemented the protein-level analysis with BioGrid 4.4. (57) curated protein-protein interaction assay data (undirected edges), and for vincristine the relevant drug target genes were selected from Ceppi et al., (2014) (58). SPOKE is hosted as a Neo4j database instance to enable efficient querying and analysis of the biological network. Cypher query language was used to extract participates in (GO terms) and perturbation assay (Compound/overexpression/knockdown affects gene) gene relationships. The allShortestPaths method in Neo4j was then used to find all shortest paths between the chromosome 12 genes and drug target genes/proteins; NR3C1/GCR_HUMAN for prednisolone, TOP2A/TOP2A_HUMAN for doxorubicin and TUBB1, MAP4, CAPG, ACTG1/TUB_HUMAN, MAP4_HUMAN, CAPG_HUMAN, ACTG_HUMAN for vincristine. The resulting network structures were visualized using Gephi. Specific rules on edge types/properties were applied to simplify the network graphs. We limited the network to a maximum 2-hops from each drug target. To only retain evidence for strong co-regulation, gene perturbation edges were removed unless the gene and drug target had at least 10 such connections. Due to the high number of "participates in" (GO term) edges associated with ACTG1, we excluded this gene from the network metric analysis step. Subsequently, network

metrics were calculated using the available functions in networkX python library. To assess the topological significance of the 73 genes from chromosome 12 and drug target genes within the network, we performed an empirical permutation test by selecting 73 genes randomly from SPOKE and repeating the shortest path calculation for 1000 iterations to generate a null distribution.

The analysis of genes from chromosome 12 that are annotated with a statistically significant effect on cell fitness based on cancer cell line CRISPR screens in DepMap (59,60) or Project Score was carried out using the Galaxy interface for oncoEnrichr (61).

## References


1. Somasundaram R, Jensen CT, Tingvall-Gustafsson J, Åhsberg J, Okuyama K, Prasad M, et al. EBF1 and PAX5 control pro-B cell expansion via opposing regulation of the Myc gene. Blood. 2021 Jun 3;137(22):3037–49.

2. Li Z, Zhao H, Yang W, Maillard M, Yoshimura S, Hsiao YC, et al. Molecular and pharmacological heterogeneity of ETV6::RUNX1 acute lymphoblastic leukemia. Nat Commun. 2025 Jan 29;16(1):1153.

3. Ewels P, Magnusson M, Lundin S, Käller M. MultiQC: summarize analysis results for multiple tools and samples in a single report. Bioinformatics. 2016 Oct 1;32(19):3047–8.

4. Di Tommaso P, Chatzou M, Floden EW, Barja PP, Palumbo E, Notredame C. Nextflow enables reproducible computational workflows. Nat Biotechnol. 2017 Apr;35(4):316–9.

5. Dobin A, Davis CA, Schlesinger F, Drenkow J, Zaleski C, Jha S, et al. STAR: ultrafast universal RNA-seq aligner. Bioinformatics. 2013 Jan 1;29(1):15–21.

6. Patro R, Duggal G, Love MI, Irizarry RA, Kingsford C. Salmon provides fast and bias-aware quantification of transcript expression. Nat Methods. 2017 Apr;14(4):417–9.

7. Love MI, Soneson C, Hickey PF, Johnson LK, Pierce NT, Shepherd L, et al. Tximeta: Reference sequence checksums for provenance identification in RNA-seq. PLOS Computational Biology. 2020 Feb 25;16(2):e1007664.

8. Robinson MD, McCarthy DJ, Smyth GK. edgeR: a Bioconductor package for differential expression analysis of digital gene expression data. Bioinformatics. 2010 Jan 1;26(1):139–40.

9. Ritchie ME, Phipson B, Wu D, Hu Y, Law CW, Shi W, et al. limma powers differential expression analyses for RNA-sequencing and microarray studies. Nucleic Acids Res. 2015 Apr 20;43(7):e47.

10. Hänzelmann S, Castelo R, Guinney J. GSVA: gene set variation analysis for microarray and RNA-Seq data. BMC Bioinformatics. 2013 Jan 16;14:7.

11. Mehtonen J, Teppo S, Lahnalampi M, Kokko A, Kaukonen R, Oksa L, et al. Single cell characterization of B-lymphoid differentiation and leukemic cell states during chemotherapy in ETV6-RUNX1-positive pediatric leukemia identifies drug-targetable transcription factor activities. Genome Med. 2020 Nov 20;12(1):99.

12. Gu Z, Eils R, Schlesner M. Complex heatmaps reveal patterns and correlations in multidimensional genomic data. Bioinformatics (Oxford, England). 2016 Sep 15;32(18):2847–9.

13. Andrews S. Babraham Bioinformatics - FastQC A Quality Control tool for High Throughput Sequence Data [Internet]. 2010 [cited 2023 Jul 31]. Available from: https://www.bioinformatics.babraham.ac.uk/projects/fastqc/

14. Chen S, Zhou Y, Chen Y, Gu J. fastp: an ultra-fast all-in-one FASTQ preprocessor. Bioinformatics. 2018 Sep 1;34(17):i884–90.

15. Freed D, Aldana R, Weber JA, Edwards JS. The Sentieon Genomics Tools - A fast and accurate solution to variant calling from next-generation sequence data [Internet]. bioRxiv; 2017 [cited 2024 Sep 5]. p. 115717. Available from: https://www.biorxiv.org/content/10.1101/115717v2

16. Picard Tools - By Broad Institute [Internet]. [cited 2023 Jul 31]. Available from: https://broadinstitute.github.io/picard/

17. Raine KM, Van Loo P, Wedge DC, Jones D, Menzies A, Butler AP, et al. ascatNgs: Identifying Somatically Acquired Copy-Number Alterations from Whole-Genome Sequencing Data. Curr Protoc Bioinformatics. 2016 Dec 8;56:15.9.1-15.9.17.

18. Chen X, Schulz-Trieglaff O, Shaw R, Barnes B, Schlesinger F, Källberg M, et al. Manta: rapid detection of structural variants and indels for germline and cancer sequencing applications. Bioinformatics. 2016 Apr 15;32(8):1220–2.

19. Rausch T, Zichner T, Schlattl A, Stütz AM, Benes V, Korbel JO. DELLY: structural variant discovery by integrated paired-end and split-read analysis. Bioinformatics. 2012 Sep 15;28(18):i333–9.

20. Lai Z, Markovets A, Ahdesmaki M, Chapman B, Hofmann O, McEwen R, et al. VarDict: a novel and versatile variant caller for next-generation sequencing in cancer research. Nucleic Acids Res. 2016 Jun 20;44(11):e108.

21. Robinson JT, Thorvaldsdóttir H, Winckler W, Guttman M, Lander ES, Getz G, et al. Integrative Genomics Viewer. Nat Biotechnol. 2011 Jan;29(1):24–6.

22. Quinlan AR, Hall IM. BEDTools: a flexible suite of utilities for comparing genomic features. Bioinformatics. 2010 Mar 15;26(6):841–2.

23. Amemiya HM, Kundaje A, Boyle AP. The ENCODE Blacklist: Identification of Problematic Regions of the Genome. Sci Rep. 2019 Jun 27;9(1):9354.

24. Garcia M, Juhos S, Larsson M, Olason PI, Martin M, Eisfeldt J, et al. Sarek: A portable workflow for whole-genome sequencing analysis of germline and somatic variants. F1000Res. 2020 Sep 4;9:63.

25. McLaren W, Gil L, Hunt SE, Riat HS, Ritchie GRS, Thormann A, et al. The Ensembl Variant Effect Predictor. Genome Biology. 2016 Jun 6;17(1):122.

26. Eisfeldt J, Vezzi F, Olason P, Nilsson D, Lindstrand A. TIDDIT, an efficient and comprehensive structural variant caller for massive parallel sequencing data. F1000Res. 2017 Jun 30;6:664.

27. Geoffroy V, Guignard T, Kress A, Gaillard JB, Solli-Nowlan T, Schalk A, et al. AnnotSV and knotAnnotSV: a web server for human structural variations annotations, ranking and analysis. Nucleic Acids Research. 2021 Jul 2;49(W1):W21–8.

28. Geoffroy V, Herenger Y, Kress A, Stoetzel C, Piton A, Dollfus H, et al. AnnotSV: an integrated tool for structural variations annotation. Bioinformatics. 2018 Oct 15;34(20):3572–4.

29. Nadeu F, Mas-de-les-Valls R, Navarro A, Royo R, Martín S, Villamor N, et al. IgCaller for reconstructing immunoglobulin gene rearrangements and oncogenic translocations from whole-genome sequencing in lymphoid neoplasms. Nat Commun. 2020 Jul 7;11:3390.

30. Brochet X, Lefranc MP, Giudicelli V. IMGT/V-QUEST: the highly customized and integrated system for IG and TR standardized V-J and V-D-J sequence analysis. Nucleic Acids Research. 2008 Jul 1;36(suppl_2):W503–8.

31. Ye J, Ma N, Madden TL, Ostell JM. IgBLAST: an immunoglobulin variable domain sequence analysis tool. Nucleic Acids Res. 2013 Jul;41(Web Server issue):W34–40.

32. Krali O, Marincevic-Zuniga Y, Arvidsson G, Enblad AP, Lundmark A, Sayyab S, et al. Multimodal classification of molecular subtypes in pediatric acute lymphoblastic leukemia [Internet]. medRxiv; 2023 [cited 2023 Aug 8]. p. 2023.03.24.23287613. Available from: https://www.medrxiv.org/content/10.1101/2023.03.24.23287613v1

33. Nordlund J, Bäcklin CL, Wahlberg P, Busche S, Berglund EC, Eloranta ML, et al. Genome-wide signatures of differential DNA methylation in pediatric acute lymphoblastic leukemia. Genome Biology. 2013 Sep 24;14(9):r105.

34. Davis S, Du P, Bilke S, Triche T, Jr, Bootwalla M. methylumi: Handle Illumina methylation data [Internet]. Bioconductor version: Release (3.17); 2023 [cited 2023 Jul 31]. Available from: https://bioconductor.org/packages/methylumi/

35. Hovestadt V, Zapatka M. conumee: Enhanced copy-number variation analysis using Illumina DNA methylation arrays [Internet]. Bioconductor version: Release (3.17); 2023 [cited 2023 Jul 31]. Available from: https://bioconductor.org/packages/conumee/

36. Cerami E, Gao J, Dogrusoz U, Gross BE, Sumer SO, Aksoy BA, et al. The cBio cancer genomics portal: an open platform for exploring multidimensional cancer genomics data. Cancer Discov. 2012 May;2(5):401–4.

37. Guo X, Zhang R, Liu J, Li M, Song C, Dovat S, et al. Characterization of LEF1 High Expression and Novel Mutations in Adult Acute Lymphoblastic Leukemia. PLoS One. 2015 May 5;10(5):e0125429.

38. Reva B, Antipin Y, Sander C. Predicting the functional impact of protein mutations: application to cancer genomics. Nucleic Acids Research. 2011 Sep 1;39(17):e118.

39. Ng PC, Henikoff S. SIFT: Predicting amino acid changes that affect protein function. Nucleic Acids Res. 2003 Jul 1;31(13):3812–4.

40. Adzhubei IA, Schmidt S, Peshkin L, Ramensky VE, Gerasimova A, Bork P, et al. A method and server for predicting damaging missense mutations. Nat Methods. 2010 Apr;7(4):248–9.

41. Ueno H, Yoshida K, Shiozawa Y, Nannya Y, Iijima-Yamashita Y, Kiyokawa N, et al. Landscape of driver mutations and their clinical impacts in pediatric B-cell precursor acute lymphoblastic leukemia. Blood Advances. 2020 Oct 23;4(20):5165–73.

42. Oshima K, Zhao J, Pérez-Durán P, Brown JA, Patiño-Galindo JA, Chu T, et al. Mutational and functional genetics mapping of chemotherapy resistance mechanisms in relapsed acute lymphoblastic leukemia. Nat Cancer. 2020 Nov;1(11):1113–27.

43. Poulard C, Kim HN, Fang M, Kruth K, Gagnieux C, Gerke DS, et al. Relapse-associated AURKB blunts the glucocorticoid sensitivity of B cell acute lymphoblastic leukemia. Proc Natl Acad Sci U S A. 2019 Feb 19;116(8):3052–61.

44. Vireo: Bayesian demultiplexing of pooled single-cell RNA-seq data without genotype reference | Genome Biology | Full Text [Internet]. [cited 2024 Sep 5]. Available from: https://genomebiology.biomedcentral.com/articles/10.1186/s13059-019-1865-2

45. Korthauer KD, Chu LF, Newton MA, Li Y, Thomson J, Stewart R, et al. A statistical approach for identifying differential distributions in single-cell RNA-seq experiments. Genome Biology. 2016 Oct 25;17(1):222.

46. systemsgenomics. systemsgenomics/ETV6-RUNX1_scRNAseq_Manuscript_2020_Analysis [Internet]. 2024 [cited 2025 Apr 4]. Available from: https://github.com/systemsgenomics/ETV6-RUNX1_scRNAseq_Manuscript_2020_Analysis

47. Benjamini Y, Hochberg Y. Controlling the False Discovery Rate: A Practical and Powerful Approach to Multiple Testing. Journal of the Royal Statistical Society: Series B (Methodological). 1995 Jan 1;57(1):289–300.

48. JM S, DN C, M S, D S. MutationTaster2: mutation prediction for the deep-sequencing age. Nature methods. 2014;11(4):361–2.

49. Schneider CA, Rasband WS, Eliceiri KW. NIH Image to ImageJ: 25 years of image analysis. Nature Methods. 2012;

50. Sobrero AF, Bertino JR. Endogenous thymidine and hypoxanthine are a source of error in evaluating methotrexate cytotoxicity by clonogenic assays using undialyzed fetal bovine serum. Int J Cell Cloning. 1986 Jan;4(1):51–62.

51. Umbach GE, Spitzer G, Ajani JA, Hug V, Thames H, Rudolph FB, et al. Role of culture conditions and exposure duration in determining sensitivity of human bone marrow progenitor cells to methotrexate. J Cancer Res Clin Oncol. 1986;111(3):273–6.

52. Simon M, Blatter J, Granzow C. Antifolate Pseudo-resistance Due to Elevated Levels of Thymidine and Hypoxanthine in a Commercial Serum Preparation. ANTICANCER RESEARCH. 2007;

53. Lindhagen E, Nygren P, Larsson R. The fluorometric microculture cytotoxicity assay. Nat Protoc. 2008;3(8):1364–9.

54. Frost BM, Nygren P, Gustafsson G, Forestier E, Jonsson OG, Kanerva J, et al. Increased in vitro cellular drug resistance is related to poor outcome in high-risk childhood acute lymphoblastic leukaemia. Br J Haematol. 2003 Aug;122(3):376–85.

55. Enblad AP, Krali O, Gezelius H, Lundmark A, Blom K, Andersson C, et al. Ex Vivo Drug Responses and Molecular Profiles of 597 Pediatric Acute Lymphoblastic Leukemia

Patients [Internet]. medRxiv; 2024 [cited 2025 Jan 14]. p. 2024.12.17.24319138. Available from: https://www.medrxiv.org/content/10.1101/2024.12.17.24319138v1

56. Morris JH, Soman K, Akbas RE, Zhou X, Smith B, Meng EC, et al. The scalable precision medicine open knowledge engine (SPOKE): a massive knowledge graph of biomedical information. Bioinformatics. 2023 Feb 9;39(2):btad080.

57. Stark C, Breitkreutz BJ, Reguly T, Boucher L, Breitkreutz A, Tyers M. BioGRID: a general repository for interaction datasets. Nucleic Acids Res. 2006 Jan 1;34(Database issue):D535-539.

58. Ceppi F, Langlois-Pelletier C, Gagné V, Rousseau J, Ciolino C, Lorenzo SD, et al. Polymorphisms of the vincristine pathway and response to treatment in children with childhood acute lymphoblastic leukemia. Pharmacogenomics. 2014 Jun;15(8):1105–16.

59. Pacini C, Dempster JM, Boyle I, Gonçalves E, Najgebauer H, Karakoc E, et al. Integrated cross-study datasets of genetic dependencies in cancer. Nat Commun. 2021 Mar 12;12(1):1661.

60. Behan FM, Iorio F, Picco G, Gonçalves E, Beaver CM, Migliardi G, et al. Prioritization of cancer therapeutic targets using CRISPR-Cas9 screens. Nature. 2019 Apr;568(7753):511–6.

61. Nakken S, Gundersen S, Bernal FLM, Polychronopoulos D, Hovig E, Wesche J. Comprehensive interrogation of gene lists from genome-scale cancer screens with oncoEnrichR. International Journal of Cancer. 2023;153(10):1819–28.
